# Modulation of Excited State Property Based on Benzo[a, c]phenazine Acceptor: Three Typical Excited States and Electroluminescence Performance

**DOI:** 10.3389/fchem.2019.00141

**Published:** 2019-03-22

**Authors:** Changjiang Zhou, Shengbing Xiao, Man Wang, Wenzhe Jiang, Haichao Liu, Shitong Zhang, Bing Yang

**Affiliations:** ^1^State Key Laboratory of Supramolecular Structure and Materials, College of Chemistry, Jilin University, Changchun, China; ^2^Institute of Theoretical Chemistry, Jilin University, Changchun, China

**Keywords:** OLED, phenazine, donor-acceptor, spin-orbit coupling, hybridization state

## Abstract

Throwing light upon the structure-property relationship of the excited state properties for next-generation fluorescent materials is crucial for the organic light emitting diode (OLED) field. Herein, we designed and synthesized three donor-acceptor (D-A) structure compounds based on a strong spin orbit coupling (SOC) acceptor benzo[a, c]phenazine (DPPZ) to research on the three typical types of excited states, namely, the locally-excited (LE) dominated excited state (CZP-DPPZ), the hybridized local and charge-transfer (HLCT) state (TPA-DPPZ), and the charge-transfer (CT) dominated state with TADF characteristics (PXZ-DPPZ). A theoretical combined experimental research was adopted for the excited state properties and their regulation methods of the three compounds. Benefiting from the HLCT character, TPA-DPPZ achieves the best non-doped device performance with maximum brightness of 61,951 cd m^−2^ and maximum external quantum efficiency of 3.42%, with both high photoluminescence quantum efficiency of 40.2% and high exciton utilization of 42.8%. Additionally, for the doped OLED, PXZ-DPPZ can achieve a max EQE of 9.35%, due to a suppressed triplet quenching and an enhanced SOC.

## Introduction

Over the past few decades, organic light-emitting diodes (OLEDs) have attracted much attention from academia to industry due to their advantages for high quality flat panel display and lighting applications (Tang and VanSlyke, 1987; Cao et al., 1999; Xiang et al., 2013; Jin et al., 2014; Yao et al., 2014; Krotkus et al., 2016; Chen et al., 2017, 2018; Liu et al., 2017a). For the cheap, metal-free pure organic fluorescent molecules, effective utilization of triplet exciton is the major issue to achieve high performance device according to the spin statistics rules in electro-excitons that are generated through the combination of hole- and electron-carriers (Atkins and Friedman, 2011; Lee et al., 2014; Chen et al., 2016; Guo F. et al., 2017). By now, three main mechanisms have been proposed to utilize the 75% electro-triplet excitons: the thermally-activated delayed fluorescence (TADF)(Uoyama et al., 2012; Zhang et al., 2014a,b; Zhang D. et al., 2015; Guo J. et al., 2017), the triplet–triplet annihilation (TTA)(Luo and Aziz, 2010; Chiang et al., 2013) and the hybridized local and charge-transfer (HLCT) state(Li et al., 2012; Yao et al., 2015; Zhang S. et al., 2015; Wang et al., 2016, 2017; Tang et al., 2018). Recently, Li et al also reported a highly efficient near-infrared OLED utilizing doublet excited state, which is a promising method for 100% electro-exciton utilization (Peng et al., 2015; Ai et al., 2018a,b). Especially, the HLCT excited state is decent for fast triplet utilization and high photoluminescent (PL) efficiency, which is a promising method for the next-generation OLED materials. Generally, the excited states of pure organic material can be mainly divided into two kinds: the locally-excited (LE) state and the charge-transfer (CT) state (Gao et al., 2016; Zhou et al., 2018a). The LE state possesses large orbital overlap between the hole and electron wave functions, which usually possesses higher oscillator strength, corresponding to a larger radiative transition probability; while the CT state exhibits separated wave functions between hole and electron, and it is usually considered of non-emissive. However, the CT excited state is in favor of harvesting triplet excitons through a narrowed energy splitting between singlet and triplet states. Thus, the HLCT excited state contains a coexistence of LE and CT characters, and simultaneous high PL efficiency with high exciton utilization in OLED is expected to achieve through rational state regulation(Li et al., 2014b; Liu et al., 2015, 2017b; Zhou et al., 2017).

**Figure 1:**
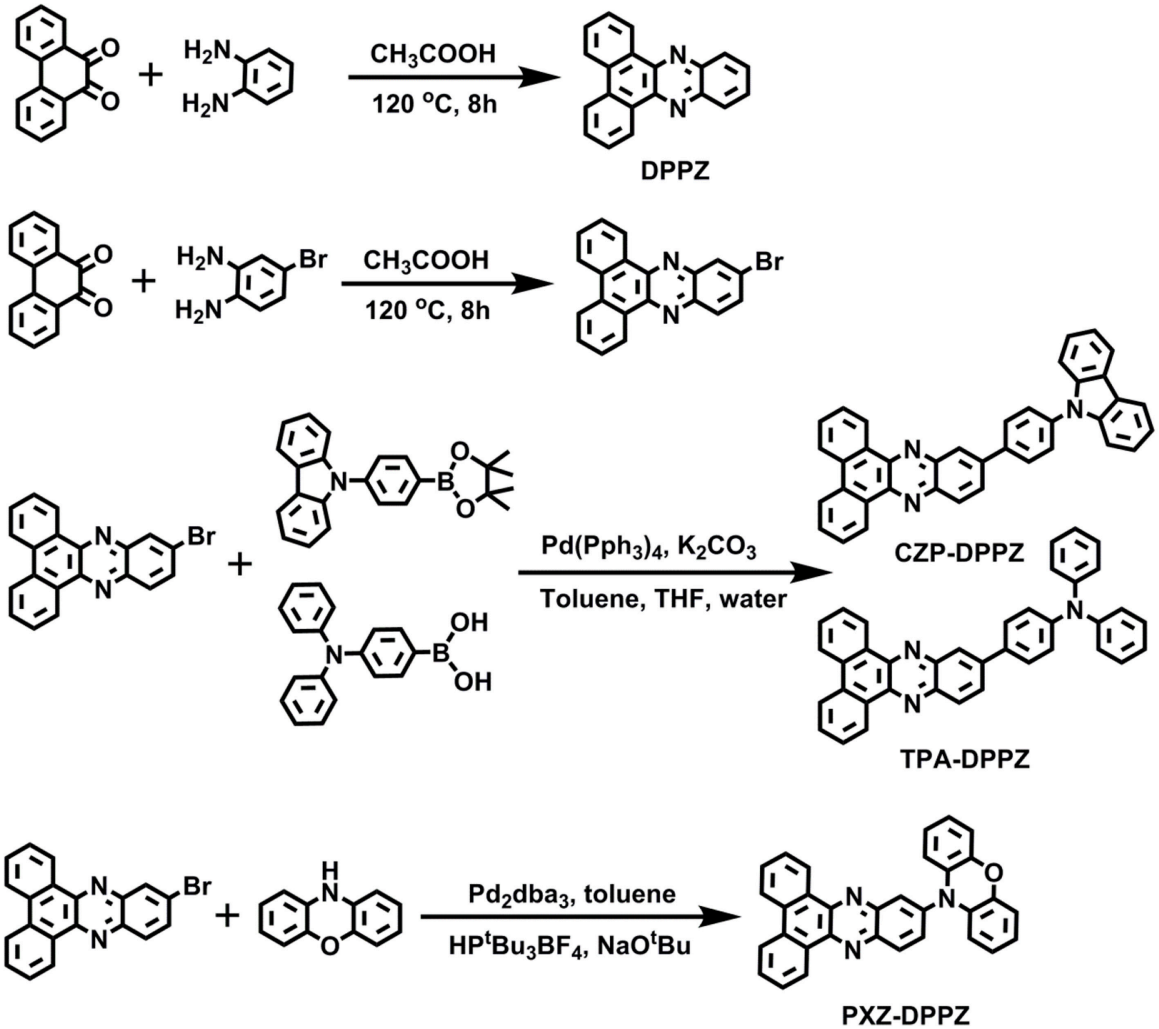
The chemical equations for the synthesis of DPPZ, CZP-DPPZ, TPA-DPPZ, and PXZ-DPPZ.

**Scheme 1:**
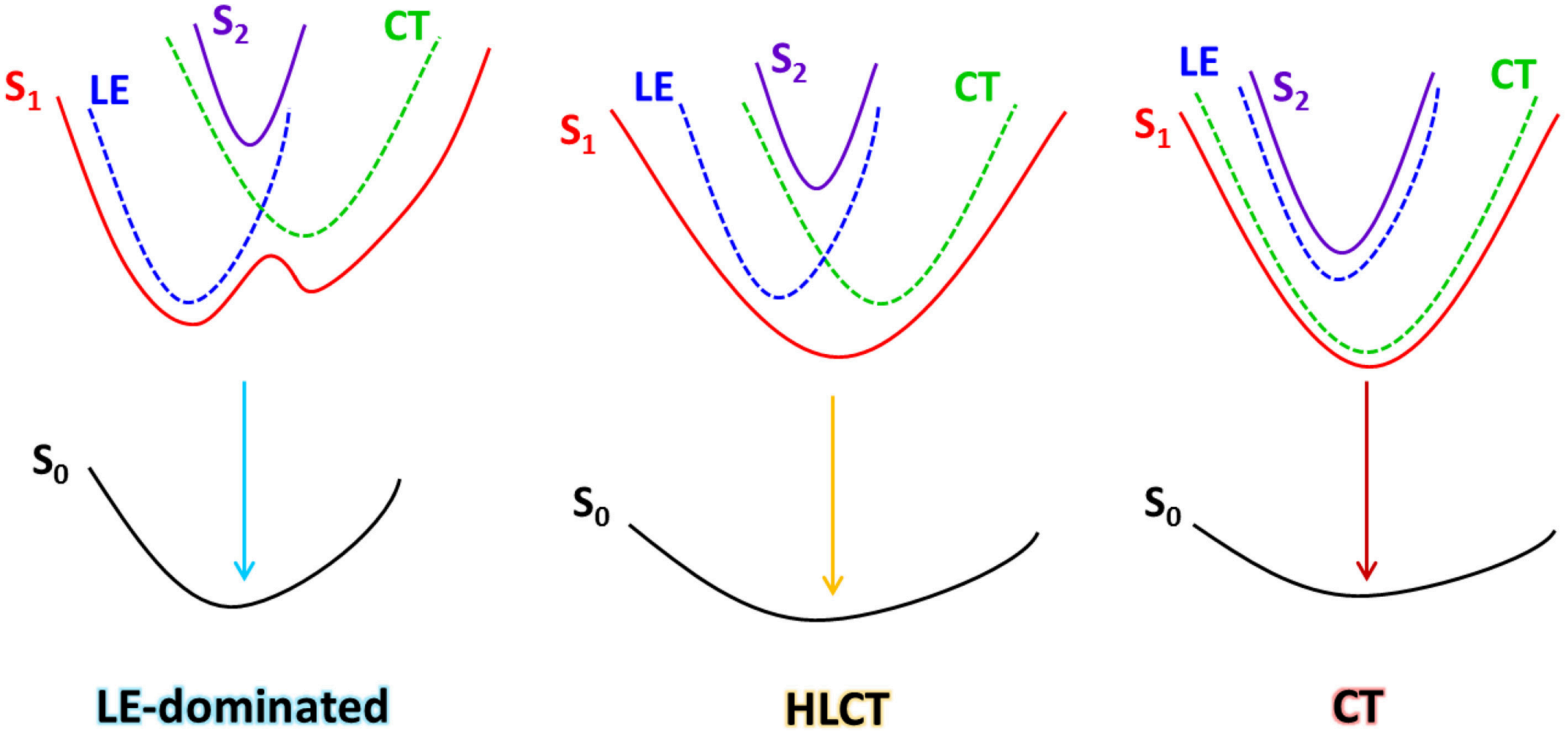
Schematic diagram of interaction between LE and CT excited states.

The explanation of high electro-triplet utilization of HLCT excited state is currently explained as going through a “hot-exciton” channel(Li et al., 2014a; Pan et al., 2014), but the essential mechanism is still under researching. Generally, the triplet utilization relies on the reversed intersystem crossing (RISC) process. Inspiring by the works on the room-temperature phosphorescence (RTP) materials, enhancing the spin-orbit coupling (SOC) can be a decent method to further accelerate the RISC for triplet utilization(Mao et al., 2015; Xu et al., 2016; Liu et al., 2018; Sun et al., 2018). Recently we have reported a singles-molecular white emissive material benzo[a, c]phenazine (DPPZ), which can realize a ternary emission of T_2_-RTP, T_1_-RTP, and S_1_-RTP (Figure 1) (Zhou et al., 2018b). We have proved that the two sp^2^-hybridized nitrogen atoms greatly contribute to the enhanced SOC in DPPZ (~10 cm^−1^) according to the El-Sayed rule(El-Sayed, 1963, 1964), and obviously, for the same reason, it can also be a suitable acceptor for the donor-acceptor (D-A) material, which is expectable for realizing decent electroluminescent (EL) performances. However, the sp^2^-hybridized nitrogen atom also causes problem in efficient emission, since the n→ π^*^ transition always performance badly in oscillator strength. Therefore, to overcome this problem, rational remolding of the excited state of DPPZ is necessary. In our previous work, introducing a proper CT excited state component to build up a HLCT excited state is an effective solution. Considering the energy-level arrangement of LE and CT origin excited states, three kinds of possible energy structures could be concluded between LE and CT states: (1) low-lying LE and high-lying CT, (2) LE lies close to CT, and (3) high-lying LE and low-lying CT (Scheme 1). Obviously, (1) and (3) are not desired model for DPPZ derivates, since the LE (originate from DPPZ) and the CT (Donor moiety → DPPZ) are neither emissive. Judging from the state-mixing principle (Equation 1 and 2):

(1) $$\begin{array}{r} \psi \left(S_{1}\right)=\psi \left(\text{LE}\right)+\lambda \times \psi \left(\text{CT}\right) \end{array}$$

(2) $$\begin{array}{r} \lambda =|\frac{\langle \psi_{LE}|H|\psi_{CT}\rangle }{E_{LE}-E_{CT}}| \end{array}$$

where λ is the mixing coefficient that represents the degree of hybridization between LE and CT, and λ is mainly determined by two factors: the energy gap of the non-adiabatic LE and CT states *E*_*LE*_ − *E*_*CT*_, and the magnitude of their interstate coupling 〈ψ_*LE*_|*H*|ψ_*CT*_〉. Obviously, it is easier to regulate *E*_*LE*_ − *E*_*CT*_ by choosing different donor units, and at the same time, its essential structure-property relationship can be further understood.

In this work, based on the DPPZ acceptor, we designed and synthesized three D-A compounds with three typical donor moieties (CZP, phenyl-carbarzole; TPA, triphenylamine; PXZ, phenoxazine), which corresponds to three typical excited states characteristics: the LE-dominated excited state (CZP-DPPZ), the HLCT (TPA-DPPZ) and CT state with TADF characters (PXZ-DPPZ). Among these three molecules, the HLCT material TPA-DPPZ obtains both high quantum efficiency of 40.2% and high exciton utilization of 42.8% in the non-doped OLED, benefiting from the HLCT character that arises the radiative transition rate and restrains the non-radiative transition, and eventually achieves the best non-doped OLED performance among the three materials with a maximum external quantum efficiency (EQE) of 3.42%. Additionally, The TADF material PXZ-DPPZ also demonstrates an efficient orange-red electro-fluorescence in doped OLED with an EQE of 9.35%.

## Materials and Methods

### Synthesis

All the reagents and solvents used for the synthesis were purchased from Aldrich or Acros and used as received. All reactions were performed under nitrogen atmosphere. The synthesis of precursor reactants can be found in our previous work.

#### 11-bromodibenzo[a, c]phenazine

A mixture of phenanthrene-9,10-dione (5 mmol) and 4-bromobenzene-1,2-diamine (5 mmol) in acetic acid (80 mL) was heated to reflux for 8 h. After cooling to room temperature, the resulting mixture was poured into ethanol (200 mL), and then filtered. The solid was washed with ethanol several times. The crude product was purified by column chromatography on silica gel (eluent: dichloromethane) and dried under vacuum to give the desired compound as a yellow solid in 90% yield (1.62 g). ^1^H NMR (500 MHz, CDCl_3_): δ 9.46–9.32 (m, 2H), 8.59 (d, *J* = 8.1 Hz, 2H), 8.56 (d, *J* = 2.0 Hz, 1H), 8.23 (d, *J* = 9.0 Hz, 1H), 7.94 (dd, *J* = 9.0, 2.1 Hz, 1H), 7.84 (t, *J* = 7.5 Hz, 2H), 7.78 (t, *J* = 7.5 Hz, 2H). MALDI-TOF MS (mass m/z): 361.4 [M(H)] ^+^.

#### 11-(4-(9H-carbazol-9-yl)phenyl)dibenzo[a, c]phenazine (CZP-DPPZ)

A mixture of 9-(4-(4,4,5,5-tetramethyl-1,3,2-dioxaborolan-2-yl)phenyl)-9H-carbazole (2.6 mmol) (Wang et al., 2016), 11-bromodibenzo[a, c]phenazine (2.0 mmol), sodium carbonate (20 mmol), toluene (15 mL), absolute alcohol (10 ml) and deionized water (10 mL), with Pd(PPh_3_)_4_ (60 mg) acting as catalyst was refluxed at 90°C for 48 h under nitrogen. After the mixture was cooled down, 40 mL water was added to the resulting solution and the mixture was extracted with CH_2_Cl_2_ for several times. The organic phase was dried over Na_2_SO_4_. After filtration and solvent evaporation, the liquid was purified by chromatography using the mixture of CH_2_Cl_2_/petroleum ether as the eluent to afford a pale green solid in 75% yield (0.78 g). ^1^H NMR (500 MHz, CDCl_3_): δ 9.59 (d, *J* = 7.9 Hz, 1H), 9.53 (d, *J* = 7.6 Hz, 1H), 8.84 (s, 1H), 8.64 (d, *J* = 7.6 Hz, 2H), 8.58 (d, *J* = 8.8 Hz, 1H), 8.30 (dd, *J* = 8.8, 1.6 Hz, 1H), 8.21 (d, *J* = 7.8 Hz, 2H), 8.15 (d, *J* = 8.3 Hz, 2H), 7.86 (ddd, *J* = 22.6, 14.0, 7.6 Hz, 6H), 7.58 (d, *J* = 8.2 Hz, 2H), 7.50 (t, *J* = 7.3 Hz, 2H), 7.36 (t, *J* = 7.4 Hz, 2H). MALDI-TOF MS (mass m/z): 522.7 [M(H)] ^+^. Anal. calcd for C_38_H_23_N_3_: C 87.50, H 4.44, N 8.06; found: C 87.13, H 4.68, N 8.16.

#### 4-(dibenzo[a, c]phenazin-11-yl)-N,N-diphenylaniline (TPA-DPPZ)

A mixture of N,N-diphenyl-4-(4,4,5,5-tetramethyl-1,3,2-dioxaborolan-2-yl)aniline (2.6 mmol), 11-bromodibenzo[a, c]phenazine (2.0 mmol), sodium carbonate (20 mmol), toluene (15 mL), absolute alcohol (10 ml) and deionized water (10 mL), with Pd(PPh_3_)_4_ (60 mg) acting as catalyst was refluxed at 90°C for 48 h under nitrogen. After the mixture was cooled down, 40 mL water was added to the resulting solution and the mixture was extracted with CH_2_Cl_2_ for several times. The organic phase was dried over Na_2_SO_4_. After filtration and solvent evaporation, the liquid was purified by chromatography using the mixture of CH_2_Cl_2_/petroleum ether as the eluent to afford a yellow-green solid in 75% yield (0.78 g). ^1^H NMR (500 MHz, CDCl_3_): δ 9.59 (d, *J* = 7.8 Hz, 1H), 9.49 (d, *J* = 6.9 Hz, 1H), 8.73 (s, 1H), 8.62 (dd, *J* = 7.8, 3.7 Hz, 2H), 8.48 (d, *J* = 8.8 Hz, 1H), 8.21 (dd, *J* = 8.9, 1.9 Hz, 1H), 7.92–7.74 (m, 6H), 7.40–7.32 (m, 4H), 7.27–7.18 (m, 6H), 7.12 (t, *J* = 7.4 Hz, 2H). MALDI-TOF MS (mass m/z): 524.2 [M(H)] ^+^. Anal. calcd for C_38_H_25_N_3_: C 87.16, H 4.81, N 8.02; found: C 86.92, H 4.75, N 8.32.

#### 10-(dibenzo[a, c]phenazin-11-yl)-10H-phenoxazine (PXZ-DPPZ)

A mixture of 10H-phenoxazine (2.0 mmol), 11-bromodibenzo[a, c]phenazine (2.2 mmol), HPtBu_3_BF_4_ (0.1 mmol), Sodium tert-butoxide (2.3 mmol), toluene (10 mL), with Pd_2_(dba)_3_ (0.06 mmol) acting as catalyst was refluxed at 110°C for 48 h under nitrogen. After the mixture was cooled down, 40 mL water was added to the resulting solution and the mixture was extracted with CH_2_Cl_2_ for several times. The organic phase was dried over Na_2_SO_4_. After filtration and solvent evaporation, the liquid was purified by chromatography using the mixture of CH_2_Cl_2_/petroleum ether as the eluent to afford a red solid in 60% yield (0.55 g). ^1^H NMR (500 MHz, CDCl_3_): δ 9.47 (dd, *J* = 8.0, 1.1 Hz, 1H), 9.44 (dd, *J* = 8.0, 1.0 Hz, 1H), 8.60 (dd, *J* = 14.0, 8.5 Hz, 3H), 8.48 (d, *J* = 2.2 Hz, 1H), 7.94–7.72 (m, 5H), 6.81 (dd, *J* = 7.9, 1.5 Hz, 2H), 6.75 (td, *J* = 7.7, 1.4 Hz, 2H), 6.66 (td, *J* = 7.7, 1.5 Hz, 2H), 6.20 (dd, *J* = 8.0, 1.3 Hz, 2H). MALDI-TOF MS (mass m/z): 462.8 [M(H)] ^+^. Anal. calcd for C_32_H_19_N_3_O: C 83.28, H 4.15, N 9.10, O 3.47; found: C 83.02, H 4.41, N 9.22, O 3.24.

## Results and Discussion

### Molecular Design

#### Structures

The structures of the compounds are illustrated in Figure 1. To gain a primary understanding of these compounds, we carried out the geometrical optimization and calculated their frontier orbital distributions (the highest occupied molecular orbital, HOMO; the lowest unoccupied molecular orbital, LUMO; in Figure S1) (Zhao and Truhlar, 2008; Frisch et al., 2009). The three compounds are all of separated HOMO and LUMO, in which the HOMOs locate on the donor moieties, whereas the LUMOs distribute on DPPZ. This bipolar molecule character is beneficial for the balanced carrier transport in OLED. Notably, the twist angle between donor and acceptor for PXZ-DPPZ is as large as 78° due to the large steric hindrance, while the dihedral angles for the other two compounds are around 35° (Table S1). Such an orthogonal molecular conformation may largely break the conjugation between PXZ and DPPZ, leading to a strong CT transition of its emissive excited state. The HOMO energy level of CZP-DPPZ, TPA-DPPZ and PXZ-DPPZ are measured as −5.58 eV, −5.26 eV, and −5.09 eV, respectively, which also implies that PXZ-DPPZ may possess an obvious CT character, while CZP-DPPZ can be a LE-like material.

#### Electron Transition Properties

**Figure 2:**
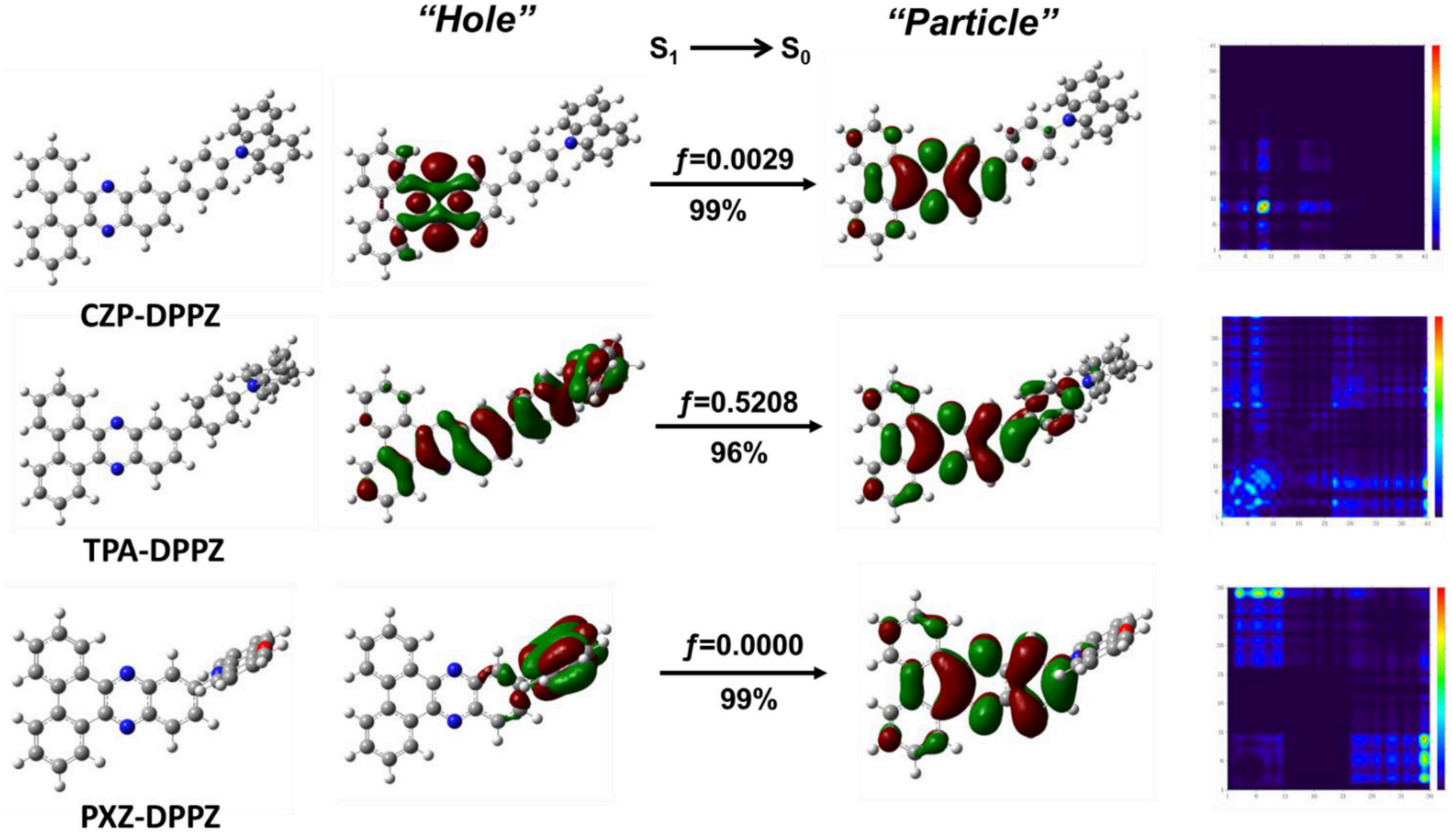
NTO and transition density matrix of the S_1_ state for CZP-DPPZ, TPA-DPPZ and PXZ-DPPZ on S_1_-geometry. Herein, *f* represents for the oscillator strength; “Hole” and “Particle” of the NTO represent for the hole- and the electron-moieties, respectively. The details of the calculation can be found in the supporting information.

To better understand the nature and character of excited states, we optimized the excited state geometries and calculated the natural transition orbital (NTO) of the emissive singlet state (S_1_→*S*_0_) for these compounds. As shown in Figure 2, the NTO “hole” and “particle” of CZP-DPPZ both localize on the DPPZ moiety, which is almost the same to NTO of pure DPPZ (Figure S2). The oscillator strength of the S_1_ state of CZP-DPPZ is 0.0029, which is only a little increasing comparing to that of DPPZ (0.0011), revealing that although certain state hybridization may occur, the S_1_ state of CZP-DPPZ mainly exhibits an obvious LE character of n→ π^*^ transition, which can result in a quenched fluorescence. On the other hand, the S_1_ state of PXZ-DPPZ shows totally vertical, separated “hole” and “particle,” assigning to an obvious CT character. And just for this reason, the oscillator strength of the S_1_ excited state is calculated to be zero, which indicates that the radiative transition in DPPZ can be very poor. Additionally, the ΔE_ST_ (E_S1_-E_T1_) of PXZ-DPPZ is estimated as 0.0238 eV, suggesting that the strong donor PXZ can potentially contribute to TADF property for PXZ-DPPZ. Therefore, neither LE-dominated nor CT-dominated is a good state regulation method of DPPZ in enhancing its emission.

Different from the nearly absolute overlap (LE) or separated (CT) character, For the S_1_ state of TPA-DPPZ, its “hole” and “particle” are partially overlapped, that is, LE and CT characters simultaneously exists in the S_1_ excited state, according with the HLCT state character. Thanks to this efficient state hybridization, the oscillator strength of TPA-DPPZ S_1_ excited state grows up to 0.5208, which is 170 times that of the LE-dominated CZP-DPPZ, indicating that TPA-DPPZ can be an emissive material by HLCT modulation. Additionally, the LE and CT compositions of the three materials can be quantitatively estimated using the transition density matrix method (Gao et al., 2016) (Figure S3) to verify our judge on the excited state categories. The LE: CT proportions of the S_1_ excited states of CZP-DPPZ, TPA-DPPZ and PXZ-DPPZ are 0.97:0.3, 0.49:0.51 and 0.13:0.87, respectively, which agrees well with their excited state properties of LE-dominated, HLCT and CT-dominated.

### Photophysical Properties

**Figure 3:**
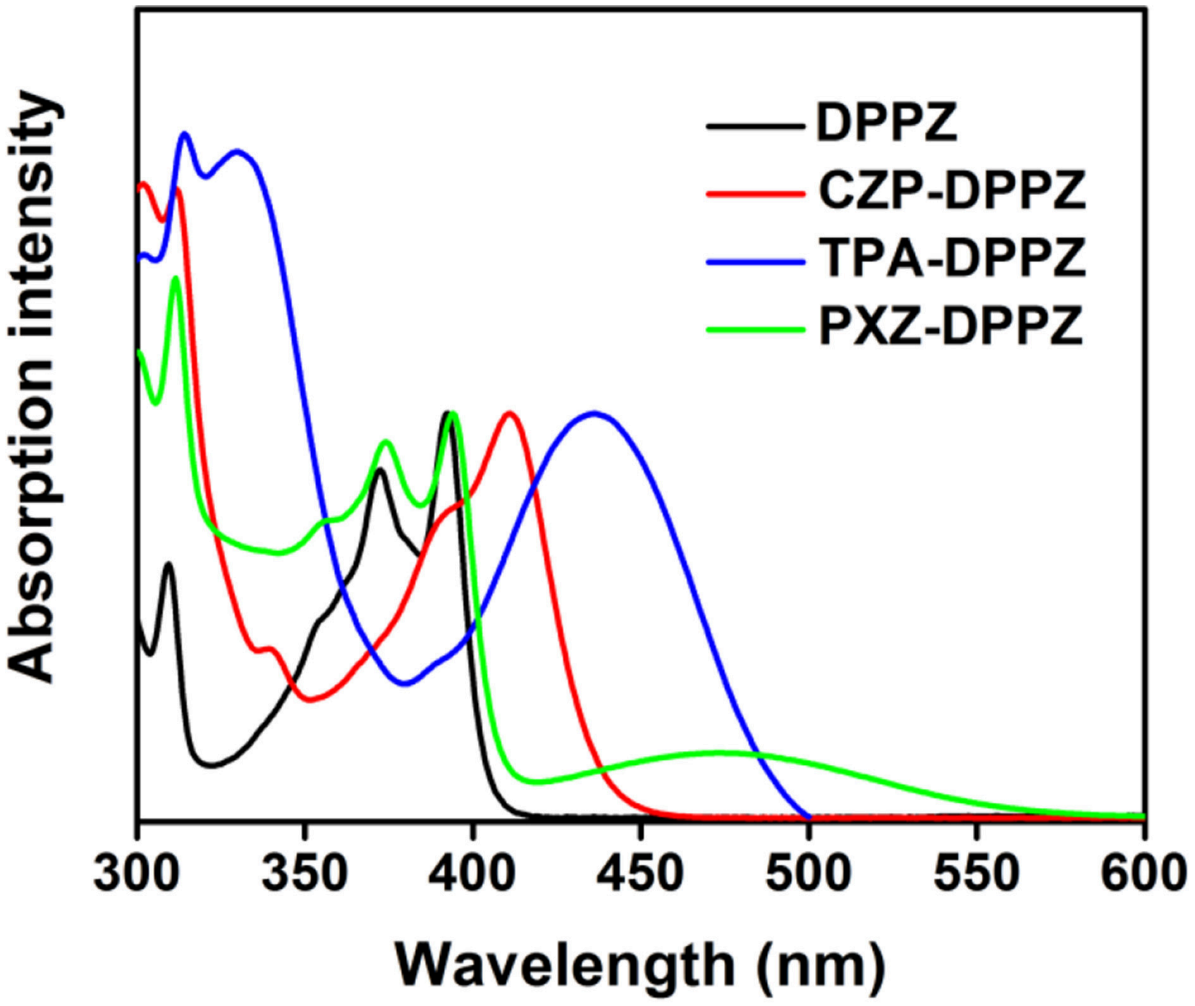
The UV-vis spectra of DPPZ and three D-A structure molecules in THF solution (the concentration is 1 × 10^−5^ mol L^−1^).

The UV spectra of these compounds were recorded in tetrahydrofuran (THF) solution (Figure 3) in reference to that of the acceptor unit DPPZ. The DPPZ unit shows vibrionic fine absorption with peaks at 371 nm and 391 nm, which is a typical character for the rigid condensed ring structure. Upon substitution of different donor moieties, characteristic absorptions can be observed to judge the excited state essences that we have predicted in the theoretical calculations. First, a single broadened absorption peak at 436 nm is observed in TPA-DPPZ, owning to the well hybridization of LE and CT. In the case of the strongest donor moiety, PXZ-DPPZ shows a new absorption with onset of 570 nm, and the original absorption peak of DPPZ is fully reserved. The newly generated, weak absorption is of a small molar absorption coefficient of below 1,000 L mol^−1^ cm^−1^ which should be ascribed to a forbidden electron transition for the small orbital overlap and large twist angle, indicating a CT-dominate absorption character, reflecting that the LE and CT components are independent, or a de-hybridized LE and CT excited state components in PXZ-DPPZ. Surprisingly, the LE-like CZP-DPPZ does not display an overlapped absorption as DPPZ or PXZ-DPPZ, instead a red-shifted spectrum with a residual fine structure is observed, which indicates that its S_1_ state is still mainly of LE state transition character of DPPZ, but certain state-hybridization of LE and CT has taken place, which may also affect its OLED performance.

**Figure 4:**
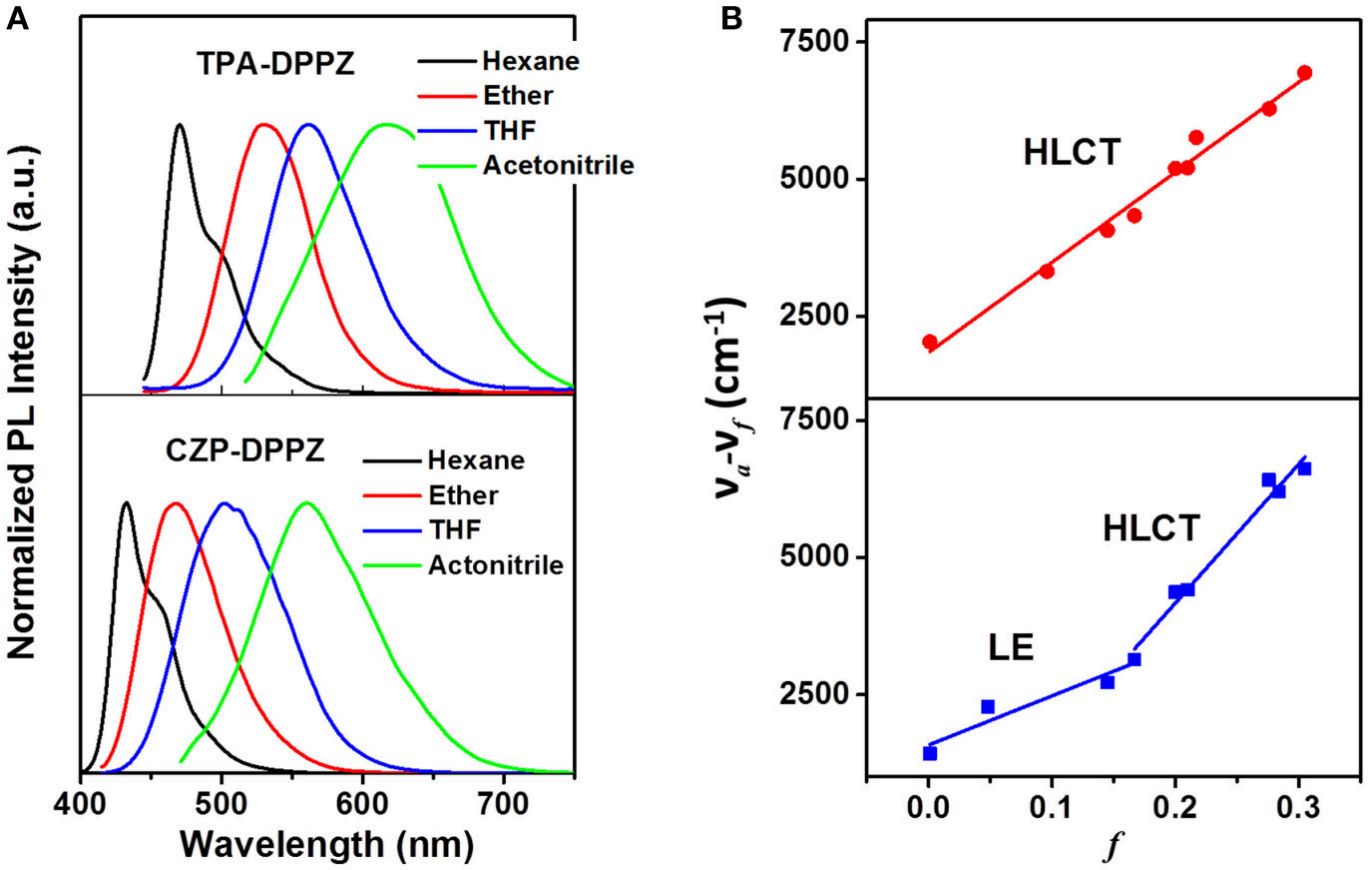
**(A)** Solvent effect on the PL spectra of CZP-DPPZ, TPA-DPPZ and PXZ-DPPZ, in which the polarity *f* of the four solvents are *f*_hexane_ =0.0012, *f*_ether_ = 0.167, *f*_THF_ = 0.210 and *f*_acetonitrile_ = 0.305. **(B)** Refined Lippert-Mataga model of CZP-DPPZ, TPA-DPPZ, and PXZ-DPPZ between Stokes shift and solvent polarity in more solvents. The details of the building of Lippert-Mataga model are in Table S2.

The solvatochromic PL measurements are then conducted for further confirm the different excited state formation of these compounds. First, CZP-DPPZ and TPA-DPPZ both exhibit red-shifted emissions in the polarity-increasing solvents, assigning to CT character (Figure 4). In their refined Lippert-Mataga model (Table S2) (Grabowski et al., 2003), obviously, CZP-DPPZ shows a two-section line with two slopes, demonstrating a non-equivalent hybridization state with two different characters of excited state, which is in accordance to its absorption: though very little, certain CT component is actually hybridized into the emissive excited state of CZP-DPPZ. The dipole moment (μ_e_) of CZP-DPPZ can be estimated to 13.57 D in low-polarity solvents and 22.74 D in high-polarity solvents, which could be assigned to a set of independent LE state and HLCT state, respectively. Different from CZP-DPPZ, in TPA-DPPZ, a good linear relation of the polarity factor *f* and the Stokes shift in all solvents is observed, corresponding to an undistinguished dipole moment of 18.41 D, assigning to the quasi-equivalent hybridization between LE and CT states. Besides, the single exponential lifetimes of TPA-DPPZ in different solvents are also evidence that the excited state is one hybridized state (Figure S4), not a simple mix of two excited states (Table S3).

**Figure 5:**
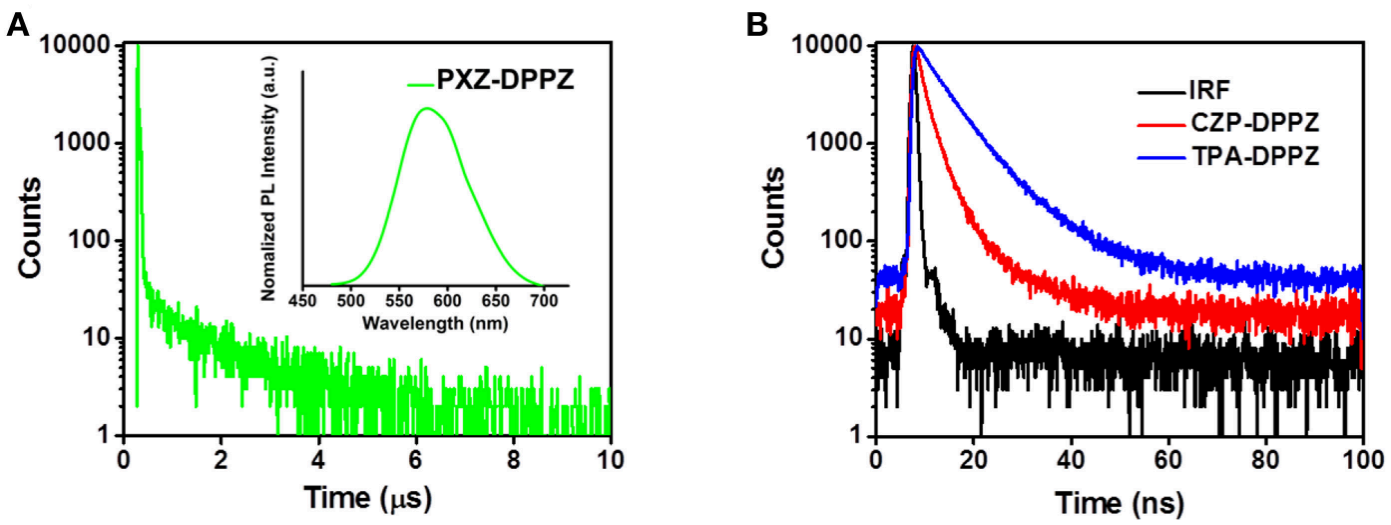
**(A)** Lifetime and PL spectra of PXZ-DPPZ and **(B)** lifetimes of CZP-DPPZ and TPA-DPPZ in doped film.

The “pure” CT compound PXZ-DPPZ demonstrates quite different PL compared to the other two compounds. We can only observe a very weak fluorescence in the lowest polarity solvent hexane with a PL peak at 566 nm (Figure S5), which is quite a red-shifted emission comparing to those of CZP-DPPZ and TPA-DPPZ, due to its obvious CT character caused by state de-hybridization. However, in higher-polarity solvents, the even strengthened CT character of PZX-DPPZ causes totally non-emissive, so that its Lippert-Mataga model cannot be built. In order to figure out its PL properties, we prepared a 5% doped film (w/w, PXZ-DPPZ in PMMA,) on neat quartz plate. As shown in Figure 5A, the doped film of PXZ-DPPZ shows an orange emission with a PL peak at 579 nm. Different from the other two compounds (Figure 5B), the CT compound PXZ-DPPZ demonstrates a typical TADF character, whose PL decay spectrum can be fitted as a bi-exponential model, where the delayed component (τ_d_) exhibits a longer lifetime of 1.3 μs in the time range of 10 μs at room temperature, and the prompt component (τ_p_) is estimated as 10.0 ns in the time range of 200 ns (Figure S6). Owning to the joint action of strong donor and orthogonal configuration, PXZ-DPPZ demonstrates a nearly zero overlap of wave functions, resulting in the small ΔE_ST_ and strong CT with TADF characters.

In addition, the photoluminescent quantum yield (PLQY) of three compounds are measured for different statuses (Solutions, doped films, and neat powder) in Table 1. As a result, the HLCT compound TPA-DPPZ keeps the highest quantum yields than others in any status, suggesting that the HLCT compound TPA-DPPZ is more promising than CZP-DPPZ and PXZ-DPPZ in non-doped OLED. Furthermore, the radiative and non-radiative transition rate constants of the three compounds are given out by theoretical calculation (Table S4) using the Molecular Materials Property Prediction Package (MOMAP) to understand the PLQY variation of the compounds (Peng et al., 2007, 2013). CZP-DPPZ displays a low radiative transition rate of 7.32 × 10^6^ s^−1^, which originate from the non-emissive n→ π^*^ character of LE state. Similarly, owing to the forbidden transition of CT state, PXZ-DPPZ exhibits the lowest k_r_ of 1.82 × 10^3^ s^−1^ and corresponding to a low PLQY. But in the case of the HLCT material TPA-DPPZ, the k_r_ largely increases to 1.23 × 10^8^ s^−1^, more importantly, its k_nr_ also shows a significant suppression comparing to the LE compound CZP-DPPZ, which is in accordance to our previous work (Zhang et al., 2013).

**Table 1:** Photoluminescence quantum yields (PLQYs) of compounds in different states.

**PLQY**	**CZP-DPPZ (%)**	**TPA-DPPZ (%)**	**PXZ-DPPZ (%)**
Hexane	12	51	12
Ether	36	97	–
THF	81	93	–
Doped film	31	91	22
Powder	23	40	11

Additionally, the seemingly non-emissive pure-CT compound PXZ-DPPZ is actually of considerable PLQY (11%). Although hard evidence has not been found yet, this phenomenon can be tentatively understood that the nuclear motion, such as the vibration and the rotation of D-A connection bond, i.e., electron-vibrational coupling (EVC) is expected to affect the emission of pure CT excited state in solid state (Chen et al., 2015; Etherington et al., 2016), which could be the basis that the TADF doped OLED is always highly efficient.

### Electroluminescence Performances

The energy levels of the frontier orbital measured by cyclic voltammetry (CV) method for the three materials are listed in Table S5. The thermal gravimetric analysis (TGA) measurements are also carried out to examine their thermal stabilities. All the three compounds exhibit good thermal stability with thermal-decomposition temperature (T_d_) over 430°C (Figure S7). The good thermal performance of these emissive materials will benefit the device stability in OLED.

**Figure 6:**
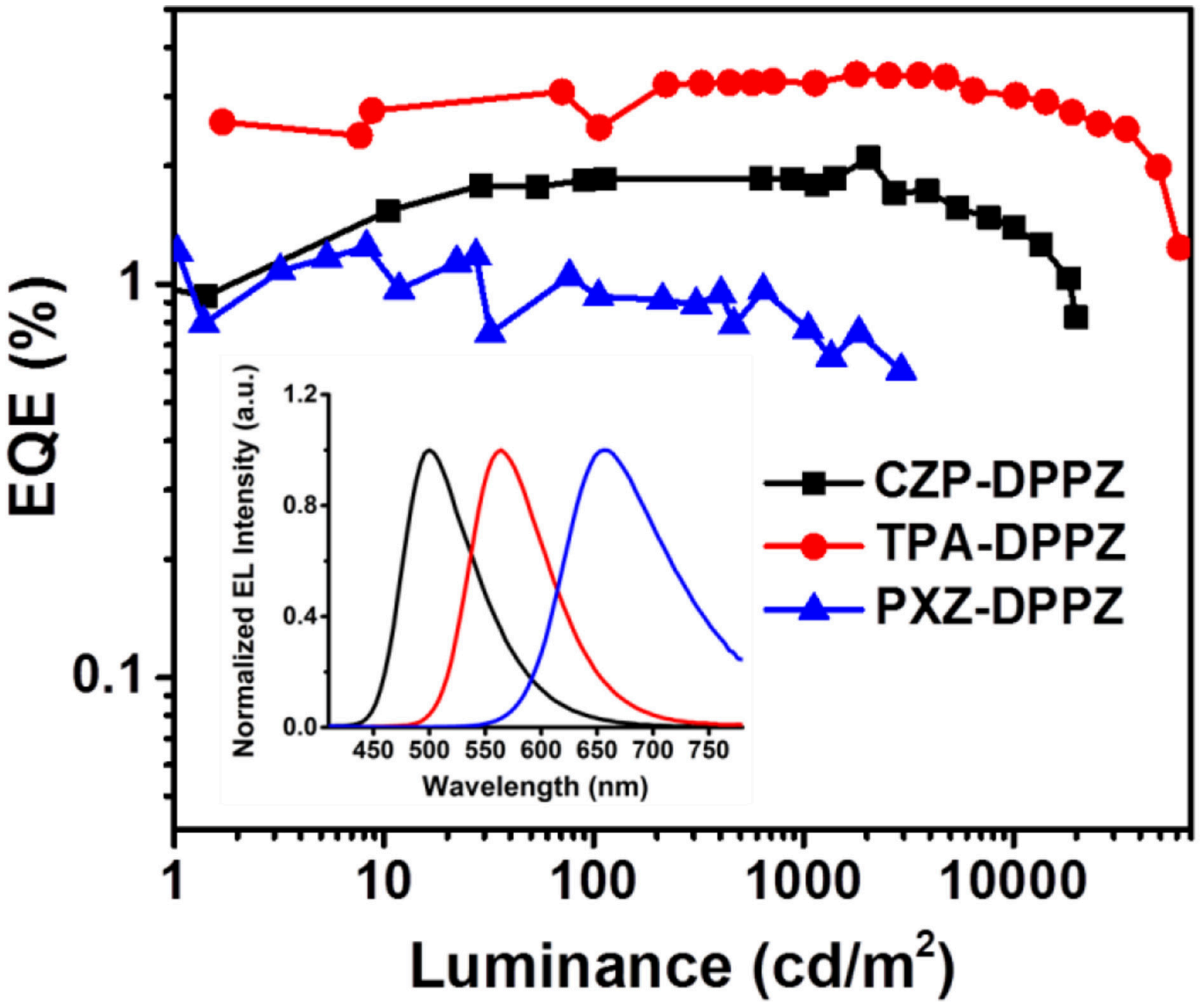
EL spectrum and external quantum efficiency vs. luminance curves of CZP-DPPZ, TPA-DPPZ, and PXZ-DPPZ devices.

The non-doped OLED using the three compounds as emitters are fabricated with typical multi-layer structure: indium tin oxide (ITO)/ hexaazatriphenylenehexacabonitrile (HATCN) (5 nm)/ 1,1′-bis(di-4-tolyl- aminophenyl)cyclohexane (TAPC) (40 nm)/4,4′,4′′-tri(N-carbazolyl)-triphenylamine (TCTA) (10 nm)/ emitter layer (20 nm)/ 1, 3, 5-tri(phenyl-2-benzimidazolyl)-benzene (TPBi) (40 nm)/ LiF (1 nm)/ Al (100 nm). Considering that the HOMO energy level of CZP is too deep (-5.58 eV), we add 4,4'-Bis(9H-carbazol-9-yl) biphenyl (CBP, HOMO = −5.91 eV) between TCTA and CZP-DPPZ as an electron-blocking layer to avoid the formation of exciplex. The device performances are summarized in Figure 6 and Table 2. The device based on CZP-DPPZ exhibits a green emission with a peak at 502 nm, and device of PXZ-DPPZ displays a red emission peaking at 656 nm (Figure S8), corresponding to the maximum external quantum efficiency (EQE) of 2.10 and 1.24%, respectively. Comparing to them, TPA-DPPZ based OLED shows significantly improved electroluminescence performance. It exhibits a lower turn-on voltage of 3.0 V, a larger maximum brightness of 61,951 cd m^−2^, reflecting quite excellent stability of device (Figure S10). What's more, it achieves a max EQE of 3.42%, which 1.5 times that of CZP-DPPZ and 3 times that of PXZ-DPPZ. This efficiency increase can be assigned to the PLQY increasing brought about by HLCT, since the electro-exciton utilization of the three materials are just similar to each other, which is the result that the three compounds are all of certain CT excited state character (Figure S9), and non-negligibly, the strong SOC between singlet and triplet from DPPZ can also be a possible structural reason in parallel comparison to the acridine based D-A compounds that we have reported before (Zhou et al., 2017, 2018a).

**Table 2:** The electroluminescence device performances of emissive materials.

**Emitter layer**	***V*_on_ (V)^a^**	**λ_max_ (nm)^b^**	***L*_max_ (cd m^−2^)^c^**	**η_LE/max_(cd A^−1^)^d^**	**η_PE/max_ (lm w^−1^)^e^**	**η_EQE/max_(%)^f^**	**η_*s*_ (%)^g^**
CZP-DPPZ	3.9	502	19,860	6.22	3.43	2.10	45.8
TPA-DPPZ	3.0	568	61,951	11.05	8.18	3.42	42.8
PXZ-DPPZ	3.6	656	2,918	0.61	0.52	1.24	49.0

a Turn-on voltage at a luminance of 1 cd m^−2^;

b Maximum peak of EL spectra;

c maximum luminance;

d Maximum luminous efficiency;

e Maximum power efficiency;

f maximum external quantum efficiency;

g *Electro-excition utilization*.

The last but not the least, in previous works, the HLCT materials perform well in the non-doped OLED but the TADF materials do well in the doped OLED, although the k_r_ of TADF materials can be very limited. To investigate this issue, we also fabricated the doped OLED for these compounds, and the data are summarized in Table 3. Although the EQE of LE-dominated compound CZP-DPPZ and the HLCT compound TPA-DPPZ are kept as the same level as their non-doped OLED, the TADF material PXZ-DPPZ demonstrates an efficient orange-red emission with an 8-fold EQE (9.35%) that of its non-doped OLED (Figure S11), benefiting from its greatly suppressed triplet quenching and the probably existed EVC interaction that can drive the pure CT excited state be emissive.

**Table 3:** Doped device performances of CZP-DPPZ, TPA-DPPZ, and PXZ-DPPZ.

**Emitter layer**	**doping (wt%)**	**V_on_(V)**	**λ_max_ (nm)**	**L_max_(cd m^−2^)**	**η_LE/max_ (cd A^−1^)**	**η_PE/max_(lm w^−1^)**	**η_EQE/max_ (%)**
CZP-DPPZ	40	3.8	492	14,030	3.59	2.38	1.46
TPA-DPPZ	10	3.6	532	15,740	12.74	10.07	3.73
PXZ-DPPZ	5	3.6	616	10,430	14.30	11.49	9.35

*The device structure is: HATCN(5 nm)/TAPA(30 nm)/TCTA(10 nm)/CBP: emitter(wt%)(20 nm)/TPBi(30 nm) /LiF(1nm)/Al(100 nm)*.

## Conclusions

In this work, to make the best use of the large SOC acceptor DPPZ, we synthesized three D-A compounds, which represent three typical excited states in fluorescent OLED: LE-dominated (CZP-DPPZ), HLCT (TPA-DPPZ), and CT (PXZ-DPPZ), and discussed the relationships between the structure and excited-state properties. Among them, the HLCT material TPA-DPPZ merits the highest k_r_ that is assigned to its enhanced oscillator strength. As a result, TPA-DPPZ performs better than the other two materials in the non-doped OLED. For the doped OLED, despite its limited k_r_, PXZ-DPPZ demonstrates a maximum EQE of 9.35%, which benefits from the greatly suppressed triplet quenching, high SOC originated from DPPZ acceptor and probably existed EVC. This work further proves that the regulation of HLCT excited state can be a decent method to design highly efficient organic functional materials, and also provides a new understanding to the structure-property relationship in fluorescent OLED.

## Data Availability

All datasets generated for this study are included in the manuscript and/or the Supplementary Files.

## Author Contributions

CZ conducted the synthesis and the theoretical calculations. CZ, SX, MW, and WJ conducted the photophysical and OLED characterizations. SZ and CZ discussed the results and wrote the manuscript. SZ, HL and BY supervised the whole work and provided the funding of this work.

### Conflict of Interest Statement

The authors declare that the research was conducted in the absence of any commercial or financial relationships that could be construed as a potential conflict of interest.
