# Supplementary material for: Modulation of Excited State Property Based on Benzo[a, c]phenazine Acceptor: Three Typical Excited States and Electroluminescence Performance
Source: Front Chem. 2019 Mar 22;7:141. doi: 10.3389/fchem.2019.00141 (PMC6439465; doi:10.3389/fchem.2019.00141)
Supplement: Supplementary file 1 [file Table_1.docx]

Supplementary Material

# Characterization and Measurements

***General information*:** The ^1^H NMR and ^13^C NMR spectra were recorded on AVANCE 500 spectrometers at 298 K by utilizing deuterated dimethyl sulfoxide (DMSO) as solvents and tetramethylsilane (TMS) as a standard. The compounds were characterized by a Flash EA 1112, CHNS-O elemental analysis instrument. The matrix-assisted laser desorption/ionization time of flight mass spectrometry (MALDI-TOF-MS) mass spectra were recorded using an AXIMA-CFR^TM^ plus instrument. Thermal gravimetric analysis (TGA) was undertaken on a PerkinElmer thermal analysis system at a heating rate of 10 °C min^-1^ and a nitrogen flow rate of 80 mL min^-1^.

***Photophysical measurements*:** UV-vis absorption spectra were recorded on a UV-3100 spectrophotometer. Fluorescence measurements were carried out with a RF-5301PC. PL efficiencies in solvents and in films were respectively measured in quartz cell and on quartz plate using an integrating sphere apparatus. The room-temperature lifetimes were measured on an Edinburgh FLS-980 with an EPL-375 optical laser.

***Lippert-Mataga model*:** The influence of solvent environment on the optical property of our compounds can be understood using the Lippert-Mataga equation, a model that describes the interactions between the solvent and the dipole moment of solute:

$$hc(\nu_{\text{a}}-\nu_{\text{f}})=hc(\nu_{\text{a}}^{0}-\nu_{\text{f}}^{0})-\frac{2(\mu_{e}-\mu_{g})^{2}}{\text{a}^{3}}f(\varepsilon,n)$$

where *f* is the orientational polarizability of solvents, *μ*_e_ is the dipole moment of excited state, *μ*_g_ is the dipole moment of ground state; *a* is the solvent cavity (Onsager) radius, *ε* and *n* are the solvent dielectric and the solvent refractive index, respectively.

***Quantum chemical calculations*:** All the density functional theory (DFT) calculations were carried out using Gaussian 09 (version D.01) package on a Power Leader cluster. The ground-state geometry was fully optimized using DFT with B3LYP hybrid functional at the basis set level of 6-31G(d, p). The excited-state geometry was optimized by time-dependent density functional theory (TD-DFT) with the B3LYP functional at the same basis set level. The absorption and emission properties were obtained using TD-M06-2X/6-31g (d, p) at the ground state and excited state geometries, respectively. The radiative and non-radiative rate constants were calculated using MOMAP (Molecular Materials Property Prediction Package) package.

***Electrochemical characterization*:** Cyclic voltammetry (CV) was performed with a BAS 100W Bioanalytical Systems, using a glass carbon disk (Φ = 3 mm) as the working electrode, a platinum wire as the auxiliary electrode with a porous ceramic wick, Ag/Ag^+^ as the reference electrode, standardized for the redox couple ferricinium/ferrocene. All solutions were purged with a nitrogen stream for 10 min before measurement. The procedure was performed at room temperature and a nitrogen atmosphere was maintained over the solution during measurements.

***Device fabrication and performances*:** The EL devices were fabricated by vacuum deposition of the materials at indium tin oxide (ITO) glass. All of the organic layers were deposited at a rate of 1.0 Å s^-1^. The cathode was deposited with LiF (1 nm) at a deposition rate of 0.1 Å s^-1^ and then capping with Al metal (100 nm) through thermal evaporation at a rate of 4.0 Å s^-1^. The electroluminescence (EL) spectra and Commission International de L’Eclairage (CIE) coordination of these devices were measured by a PR650 spectra scan spectrometer. The luminance-current density-voltage characteristics were recorded simultaneously with the measurement of the EL spectra by combining the spectrometer with a Keithley model 2400 programmable voltage-current source. All measurements were carried out at room temperature under ambient conditions.

***Calculation of exciton utilization*:** The exciton utilization efficiency (η_s_) can be evaluated according to following equation:

$$\eta_{EQE}=\gamma\times\eta_{s}\times\eta_{PL}\times\eta_{out}$$

Where η_EQE_ is the external quantum efficiency; γ is the recombination efficiency of injected holes and electrons (∼100%); η_s_ is the exciton utilization efficiency; η_PL_ is the PL efficiency of the emitter layer and η_out_ is the light out-coupling efficiency (∼20%).

# Supplementary Figures and Tables

## Supplementary Figures


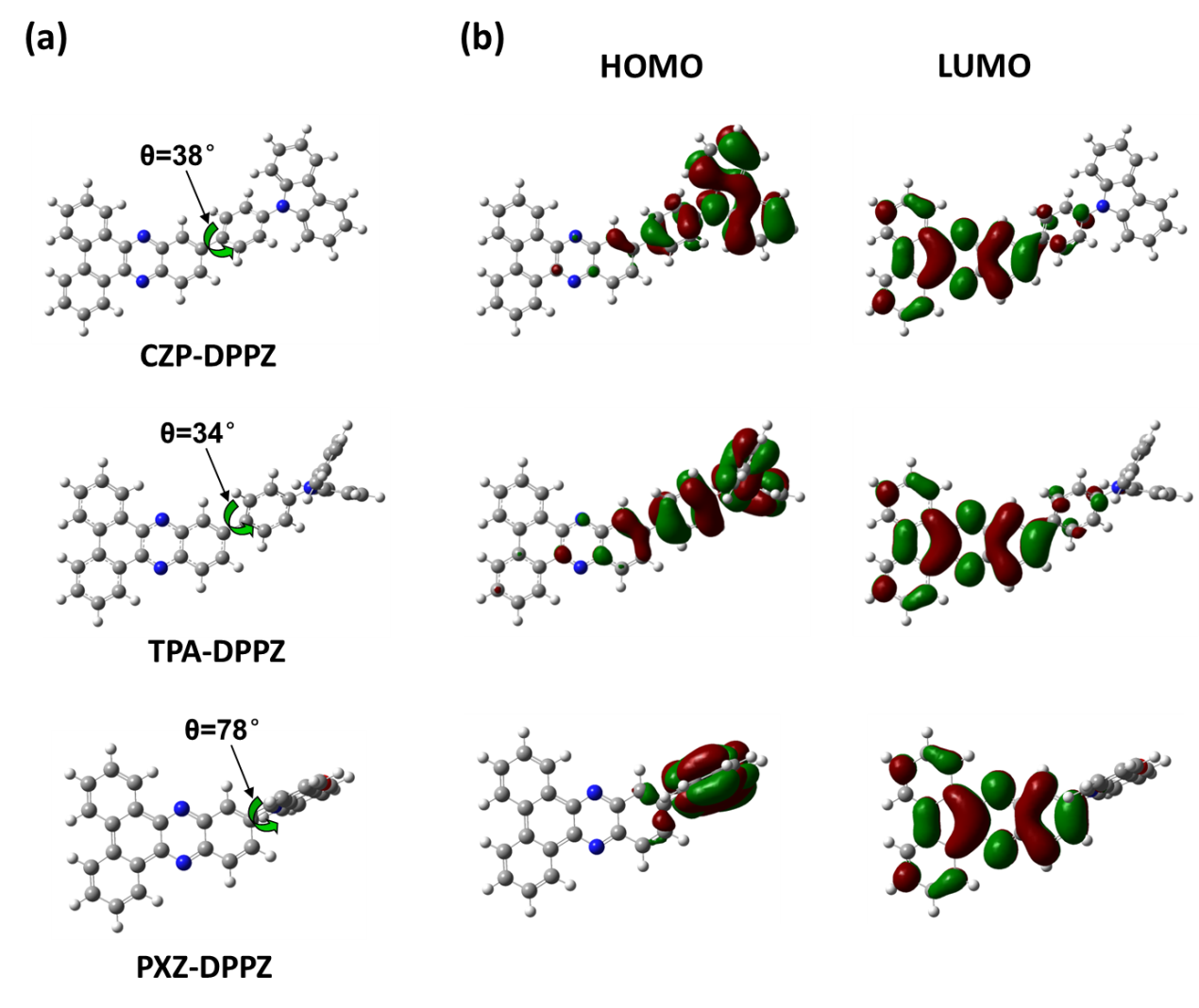


**Figure S1** The frontier molecular orbitals (HOMO and LUMO) in the ground state of DPPZ derivatives.


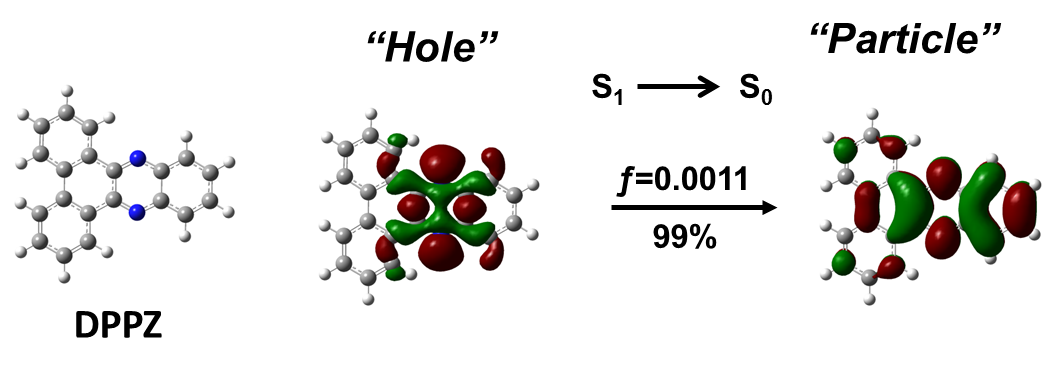


**Figure S2** NTO of the S_1_ → S_0_ transition for DPPZ at S_1_-geometry.


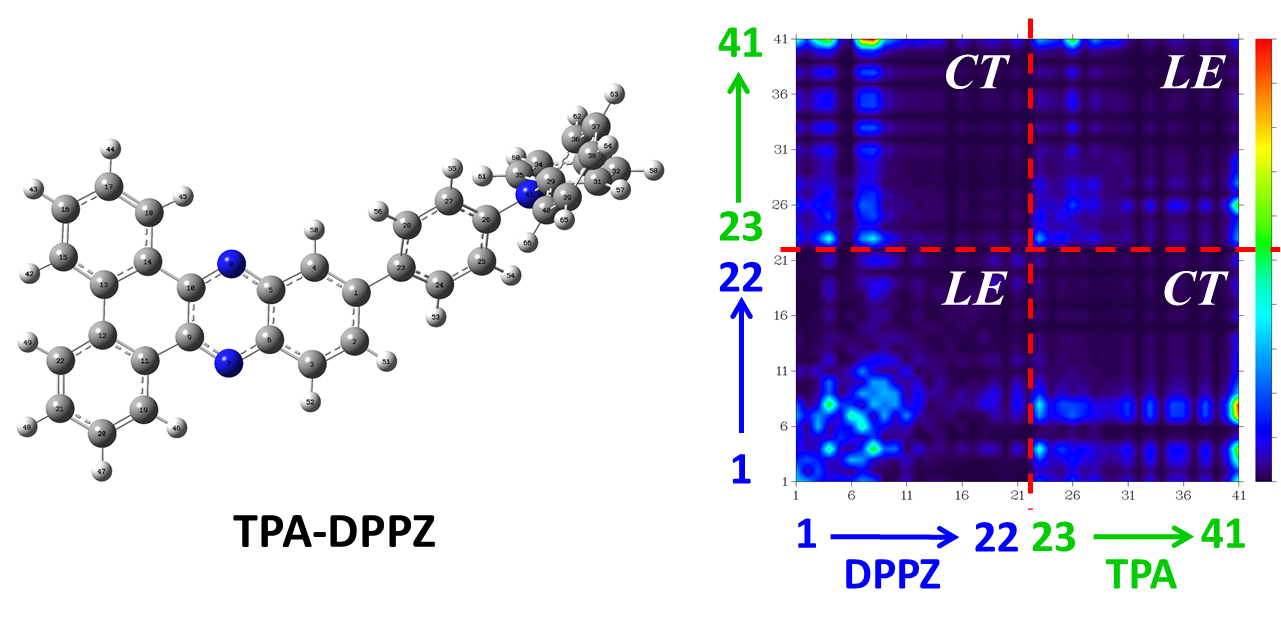


**Figure S3** Details of the transition density matrix calculation.

Take TPA-DPPZ as an example, we order the atoms from acceptor DPPZ (1-22) to donor TPA. Since hydrogen atoms usually have little contribution to the transitions we are interested in, they are labeled at the latest and usually ignored by default in TDM calculations. According to our labeling method, we divide the electron-hole pair into LE and CT areas: the diagonal part represents the LE component of monomer, while the off-diagonal region denotes the intermolecular charge transfer component from one monomer to the other. We evaluate the contents of them by using the equations below:

$$\begin{matrix} R_{LE}=\frac{M_{LE}}{M_{LE}+M_{CT}} \\ R_{CT}=\frac{M_{CT}}{M_{LE}+M_{CT}} \end{matrix}$$

In the above equations, *R_LE_*, *R_CT_* represent the ratios of LE and CT in certain excited state, *M_LE_*, *M_CT_* represent the sum of the matrix elements in LE and CT areas respectively. In this way, the LE and CT proportion of each structure could be directly calculated according to the calculus of matrix elements.

|  | CZP-DPPZ | TPA-DPPZ | PXZ-DPPZ |
| --- | --- | --- | --- |
| LE | 1.373822 | 0.660959 | 0.195562 |
| CT | 0.040694 | 0.669469 | 1.315088 |


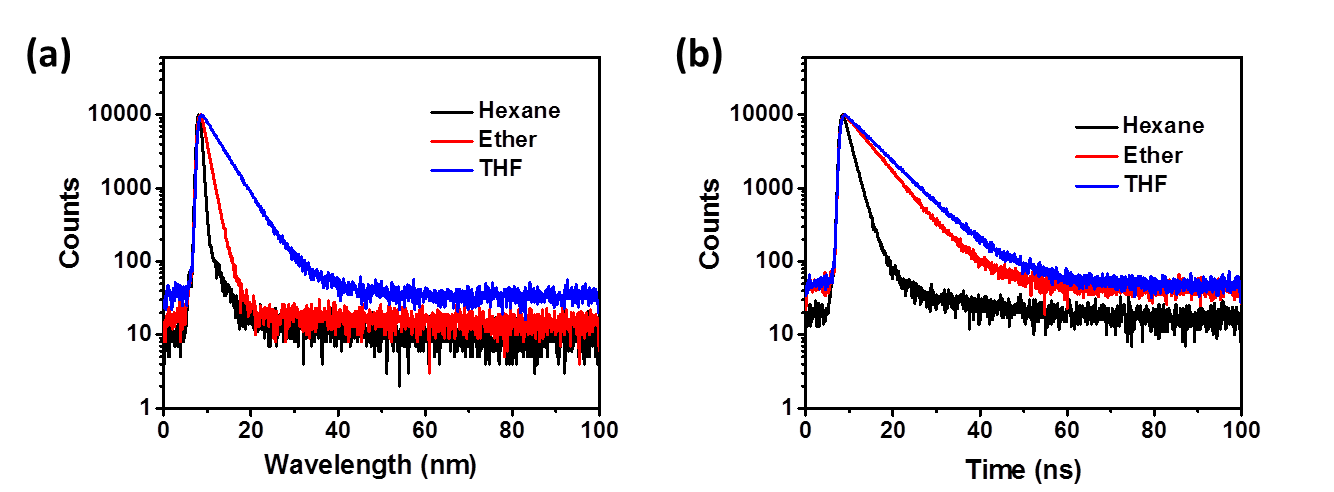


**Figure S4** Lifetime measurements of CZP-DPPZ **(a)** and TPA-DPPZ **(b)** in different solvents. For weak emission, other lifetimes are not given out.


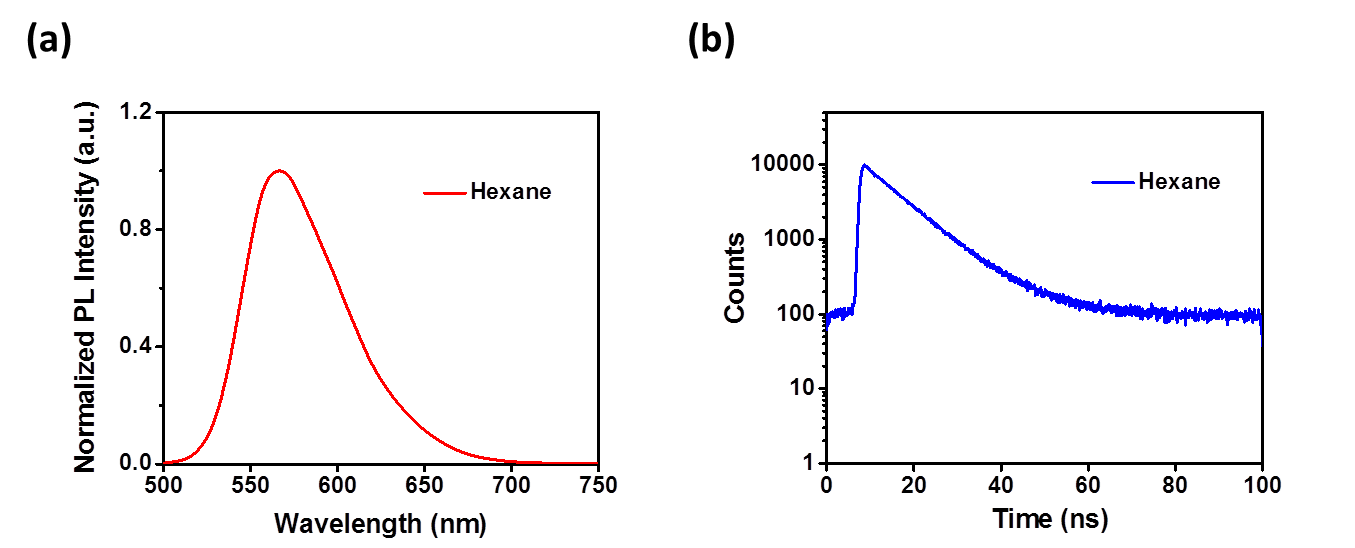


**Figure S5** PL spectrum **(a)** and lifetime **(b)** of PXZ-DPPZ in hexane solvent.


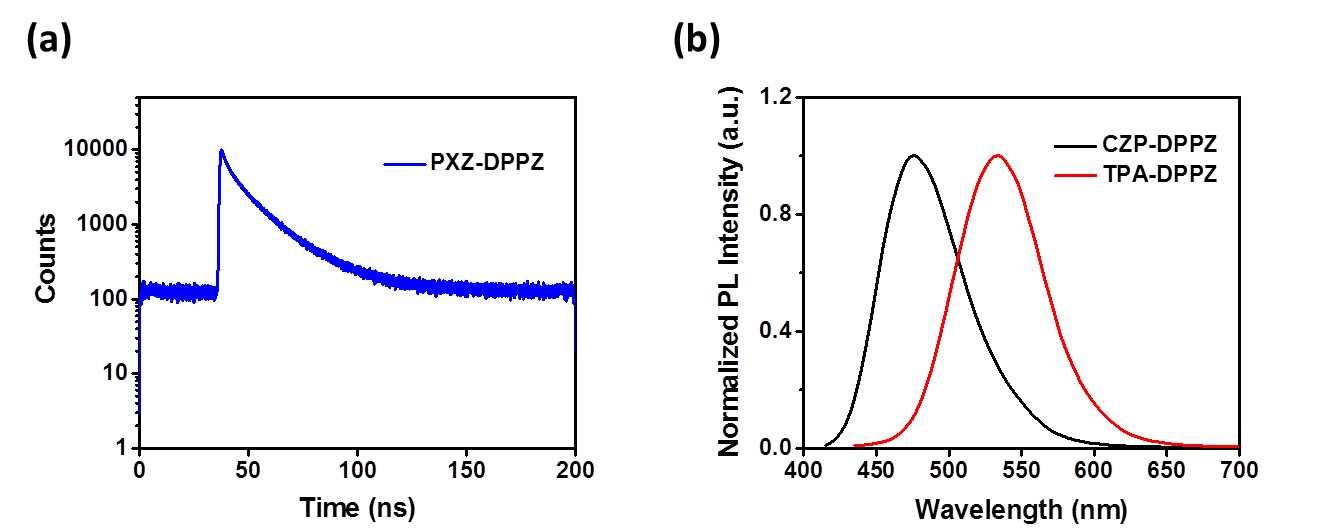


**Figure S6** Prompt lifetime **(a)** of PXZ-DPPZ at the range of 200 ns and PL spectra **(b)** of CZP-DPPZ and TPA-DPPZ in doped film.


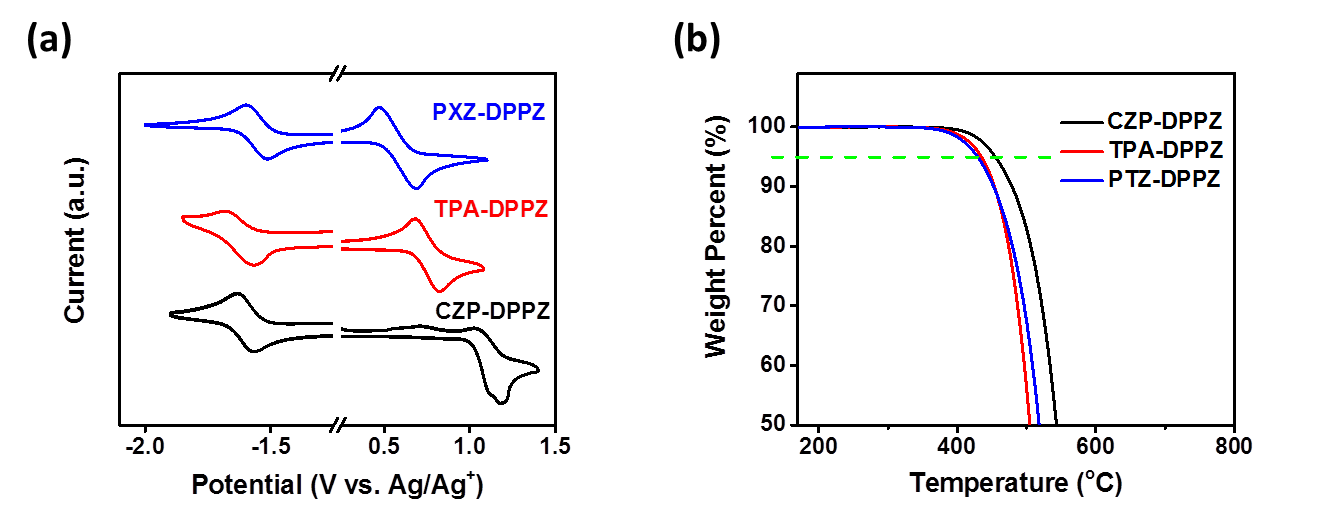


**Figure S7** The cyclic voltammetry (CV) curves **(a)** and thermal gravimetric analysis (TGA) **(b)** of CZP-DPPZ, TPA-DPPZ and PTZ-DPPZ.


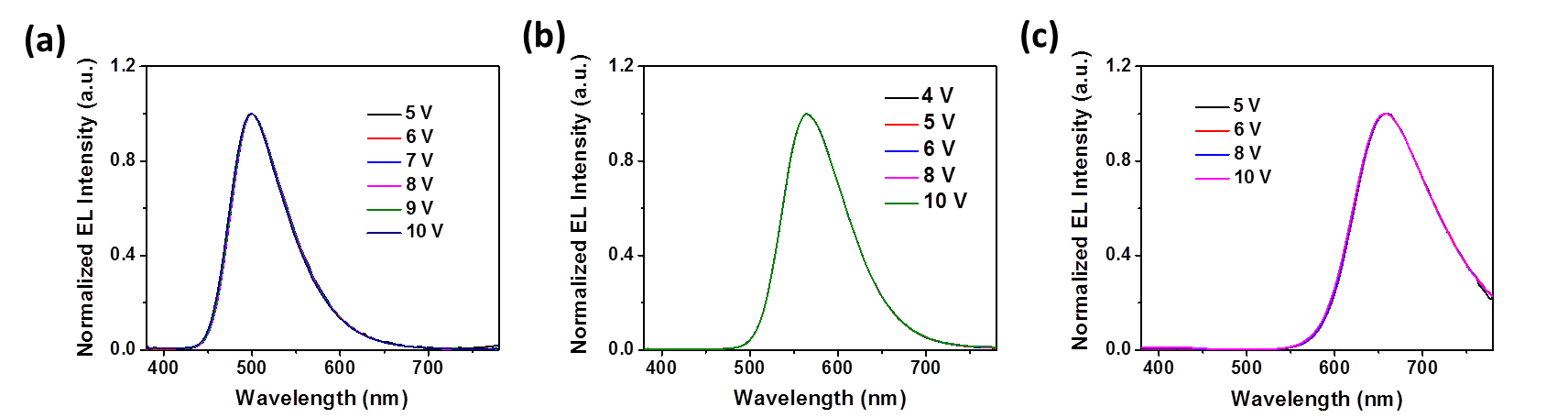


**Figure S8** The EL spectra of CZP-DPPZ **(a)**, TPA-DPPZ **(b)** and PTZ-DPPZ **(c)** based OLED at different driving voltages.


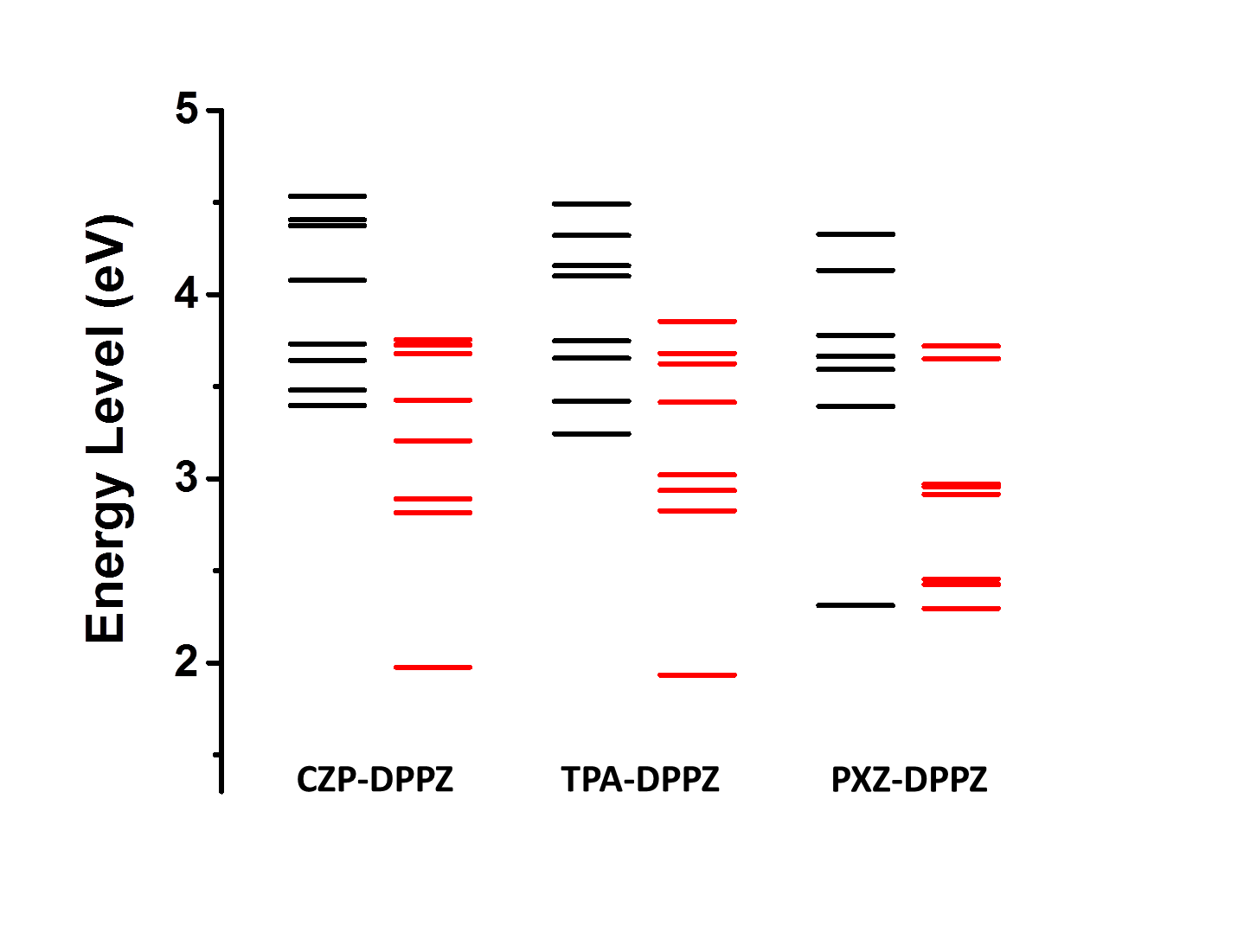


**Figure S9** The energy landscape for singlet and triplet excited states of CZP-DPPZ, TPA-DPPZ and PXZ-DPPZ.


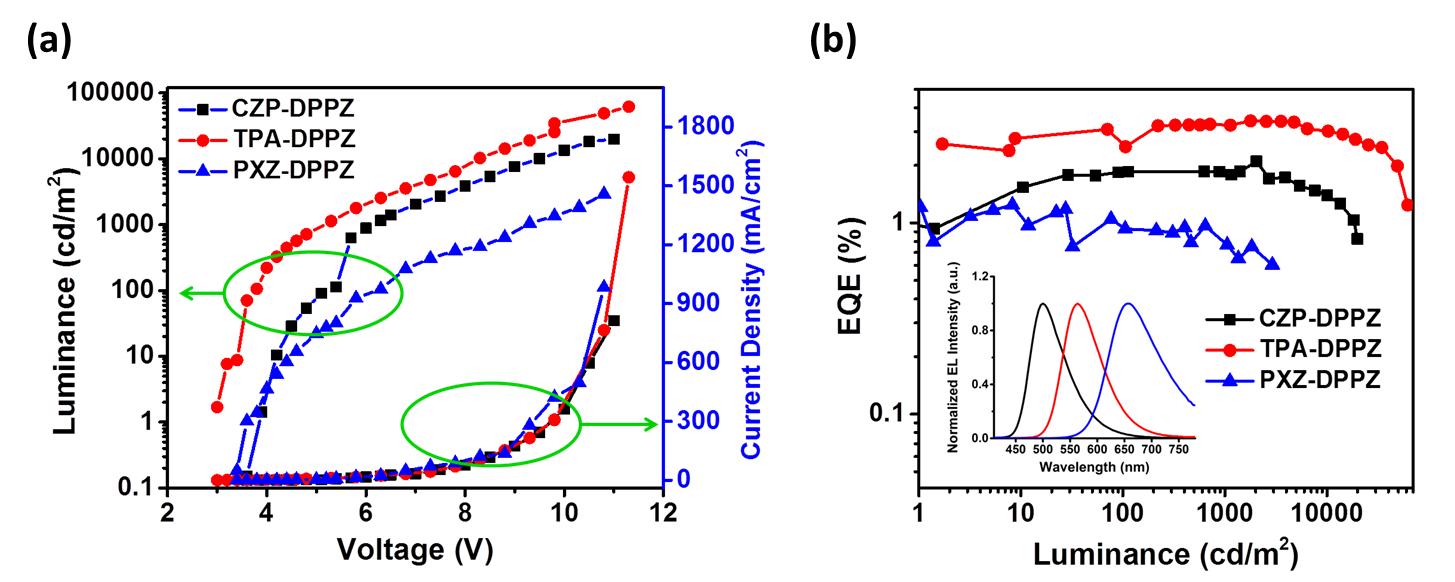


**Figure S10 (a)** Current density-voltage-brightness characterisics (J-V-L curves) and **(b)** EL spectrum and external quantum efficiency versus luminance curves of non-doped OLEDs based on CZP-DPPZ, TPA-DPPZ, and PXZ-DPPZ.


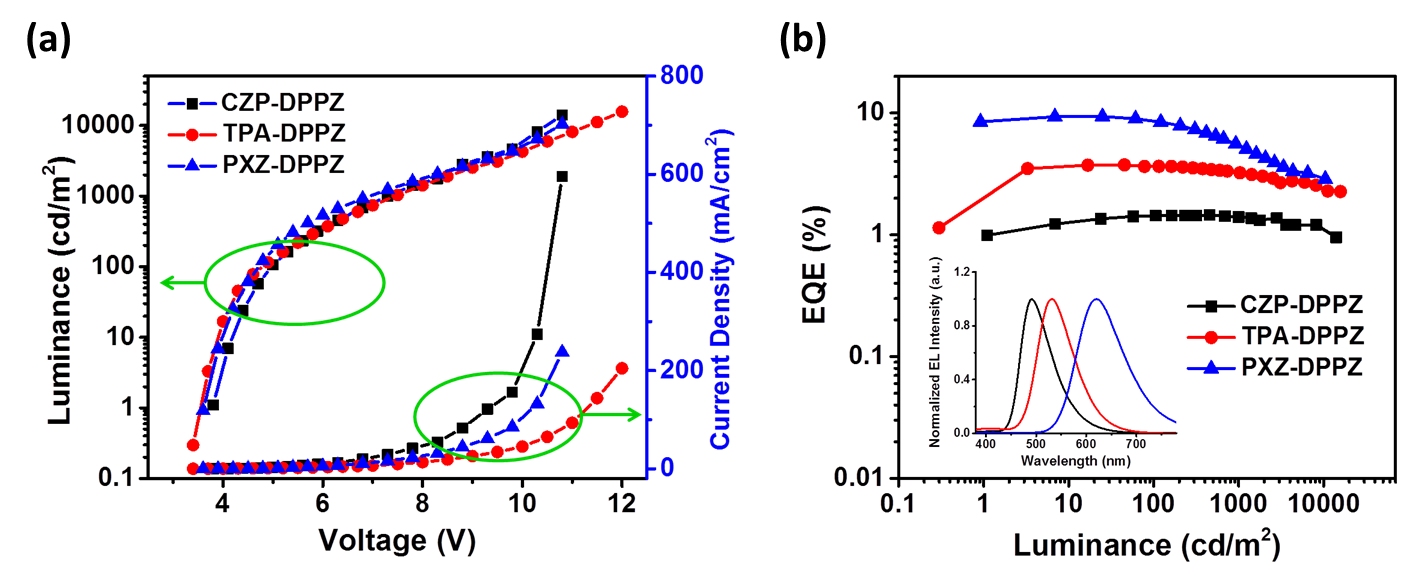


**Figure S11 (a)** Current density-voltage-brightness characterisics (J-V-L curves) and **(b)** EL spectrum and external quantum efficiency versus luminance curves of doped OLEDs based on CZP-DPPZ, TPA-DPPZ, and PXZ-DPPZ.

## Supplementary Tables

**Table S1** The ground state and excited state geometries of DPPZ derivatives.

| Molecules | Ground state | Excited state |
| --- | --- | --- |
| 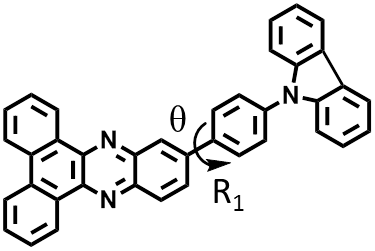  **CZP-DPPZ** | 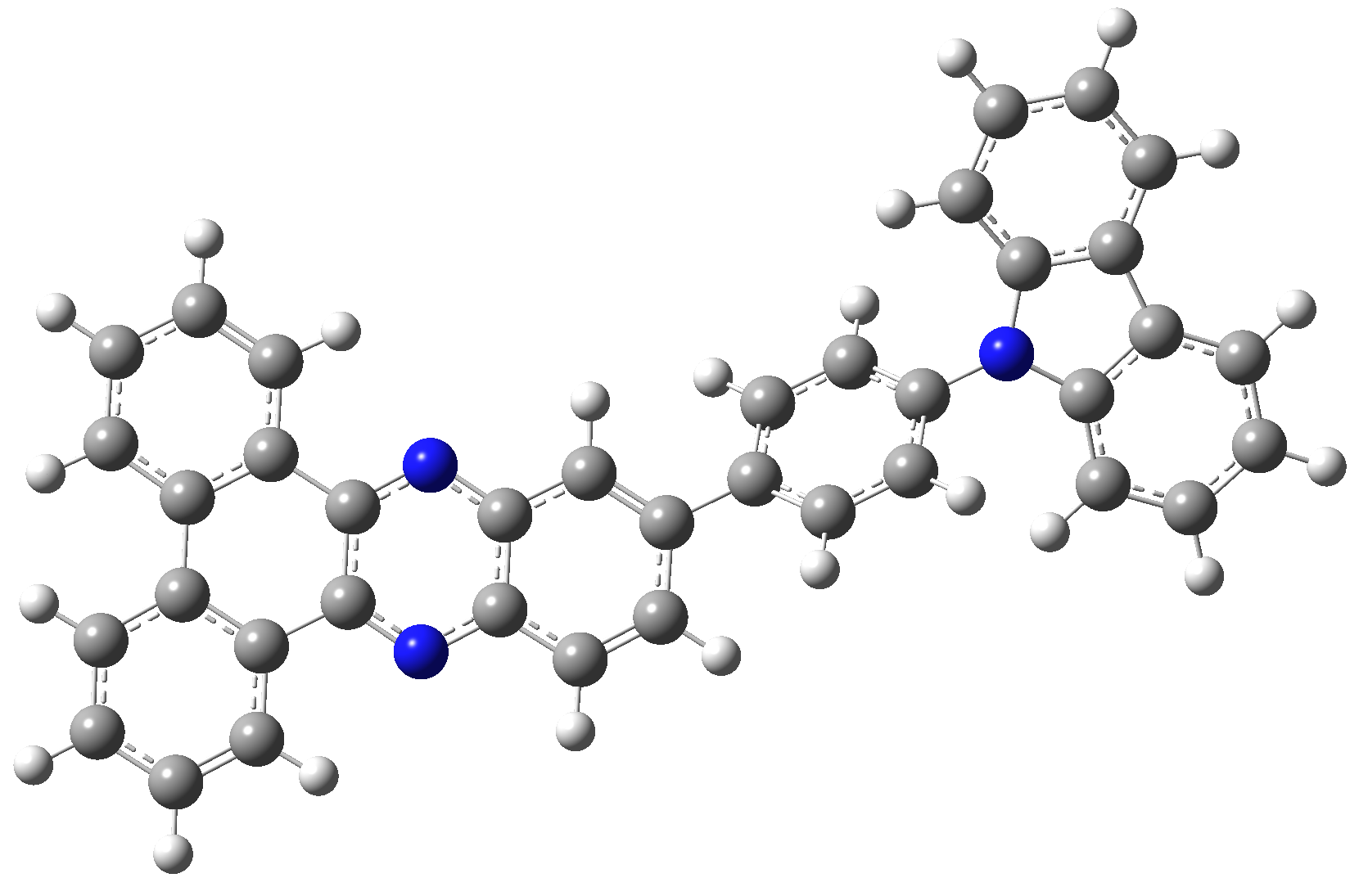 | 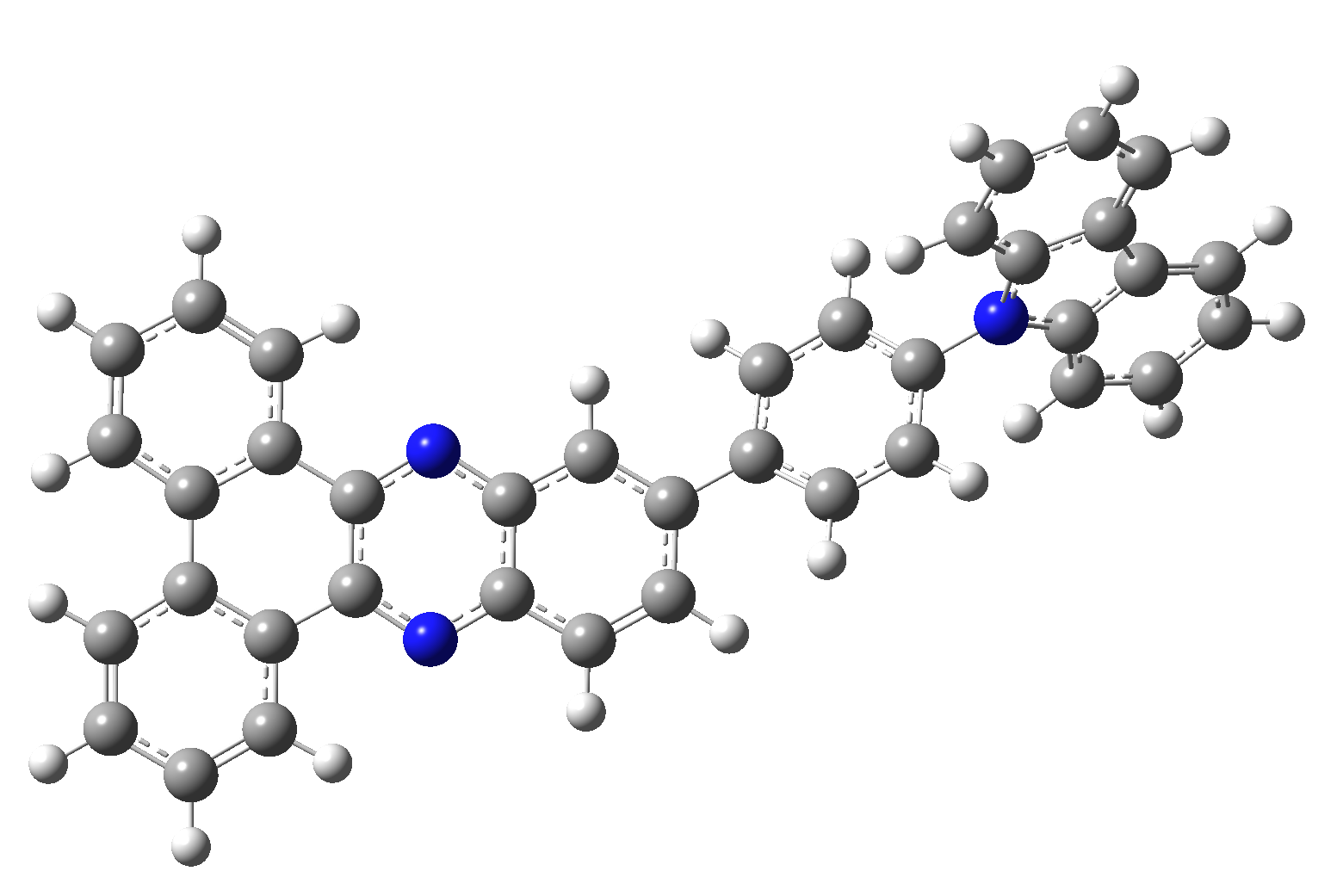 |
| Optimized geometry parameters | R_1_=1.4829Å;  θ=36.12˚; | R_1_=1.4735Å;  θ=28.52˚; |
| 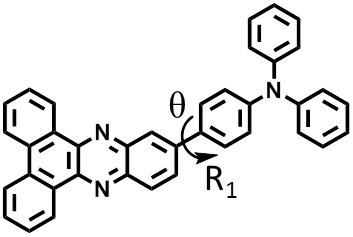  **TPA-DPPZ** | 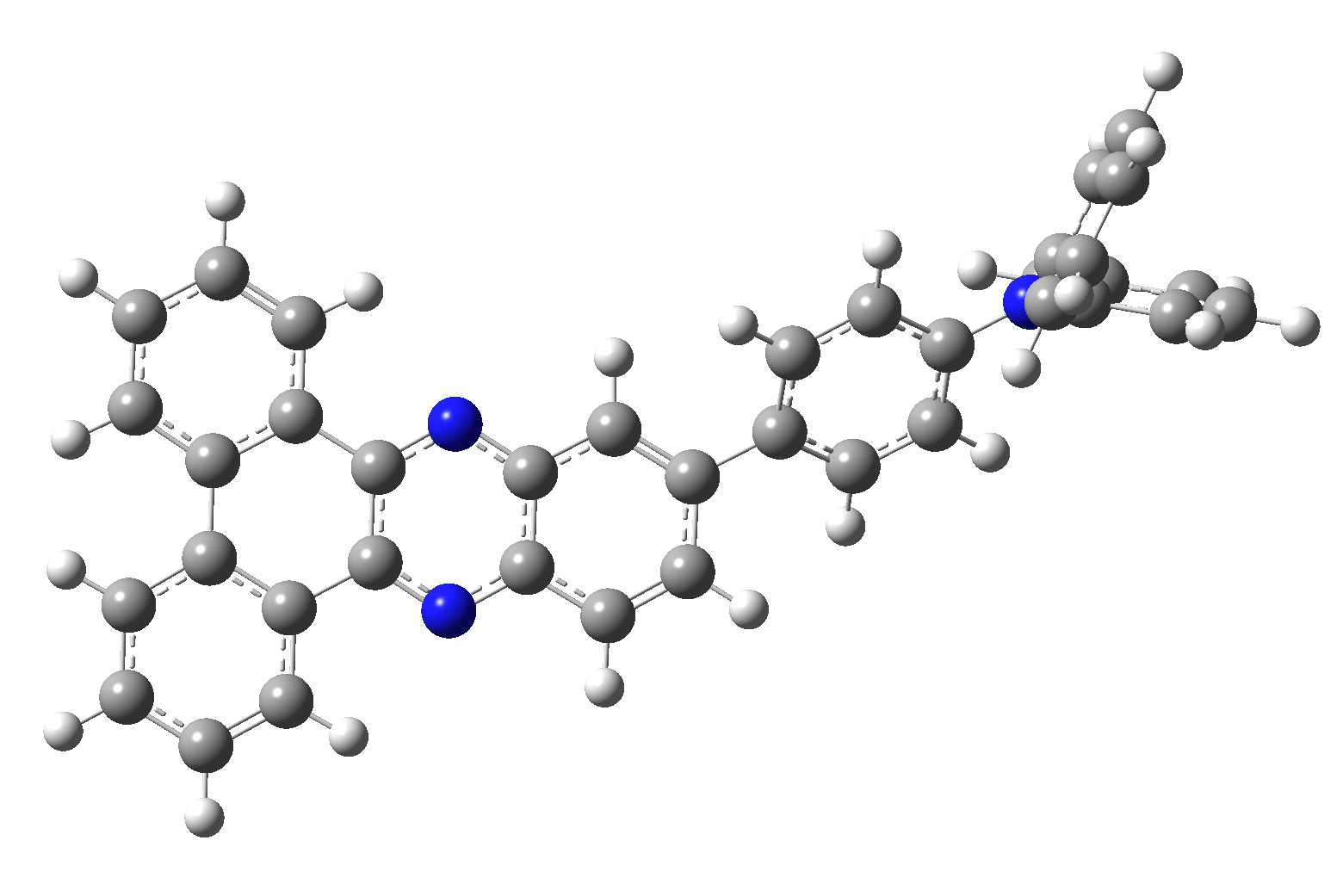 | 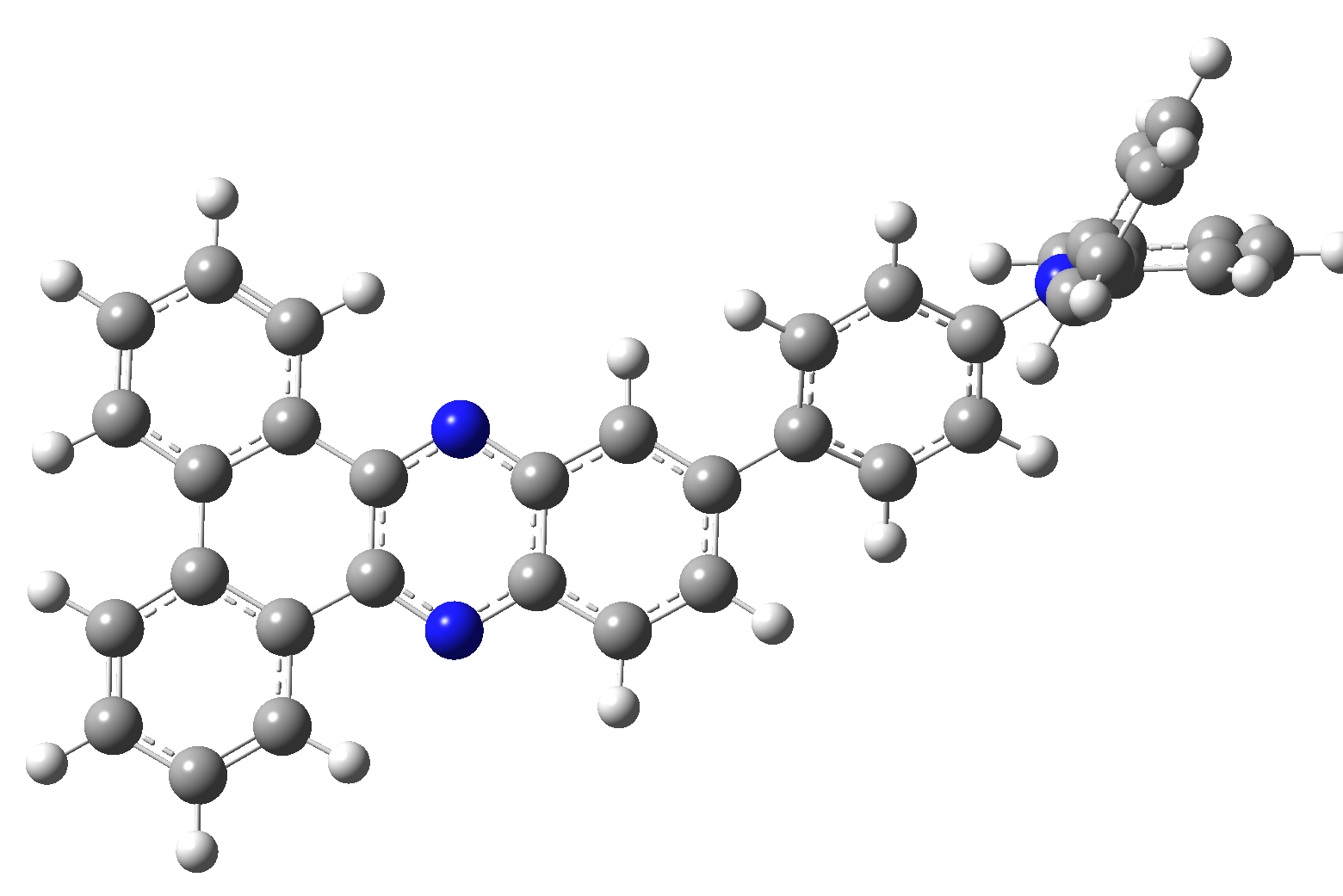 |
| Optimized geometry parameters | R_1_=1.4815Å;  θ=34.28˚; | R_1_=1.4817Å;  θ=35.08˚; |
| 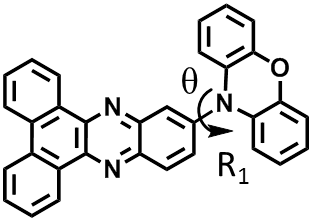  **PXZ-DPPZ** | 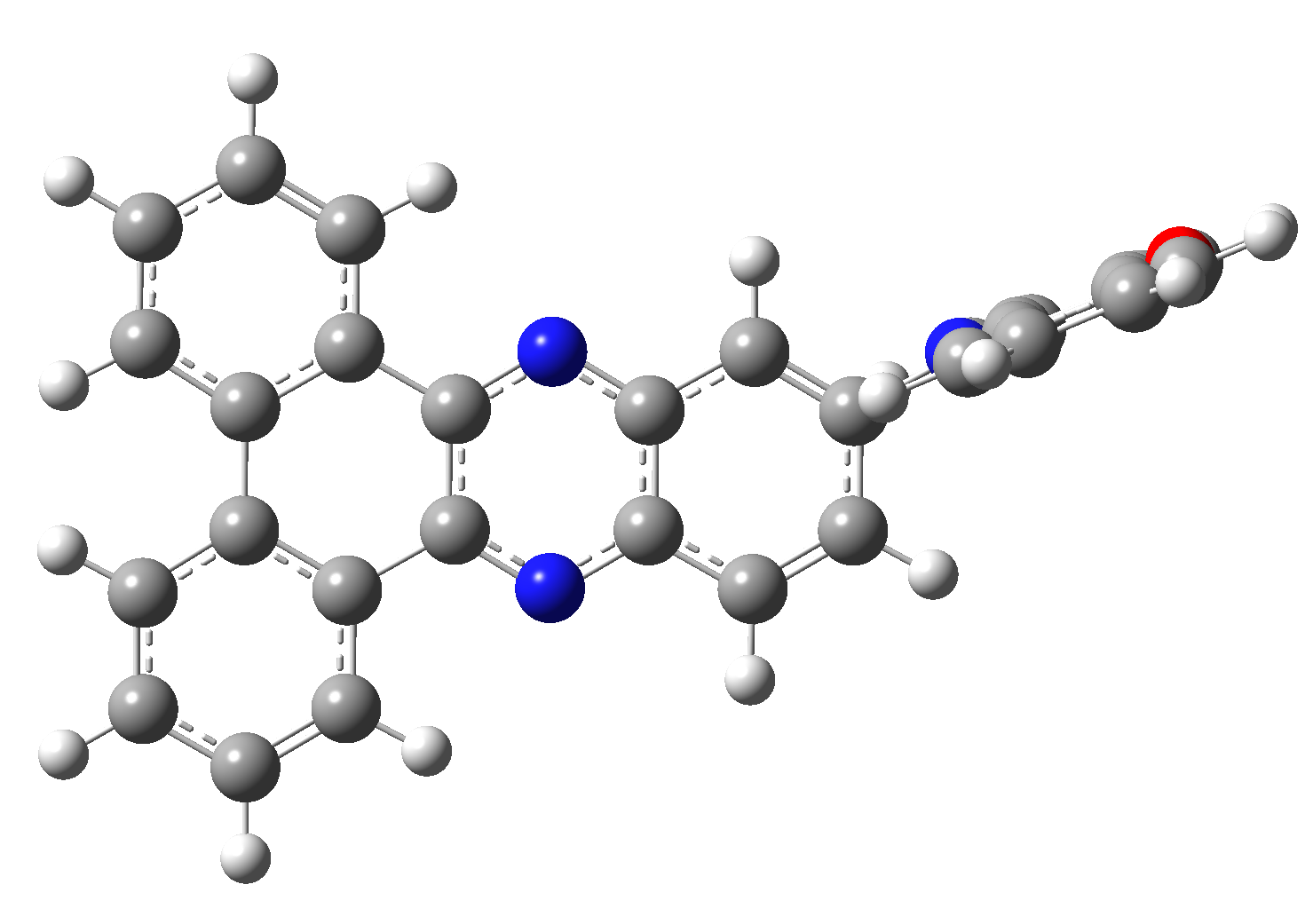 | 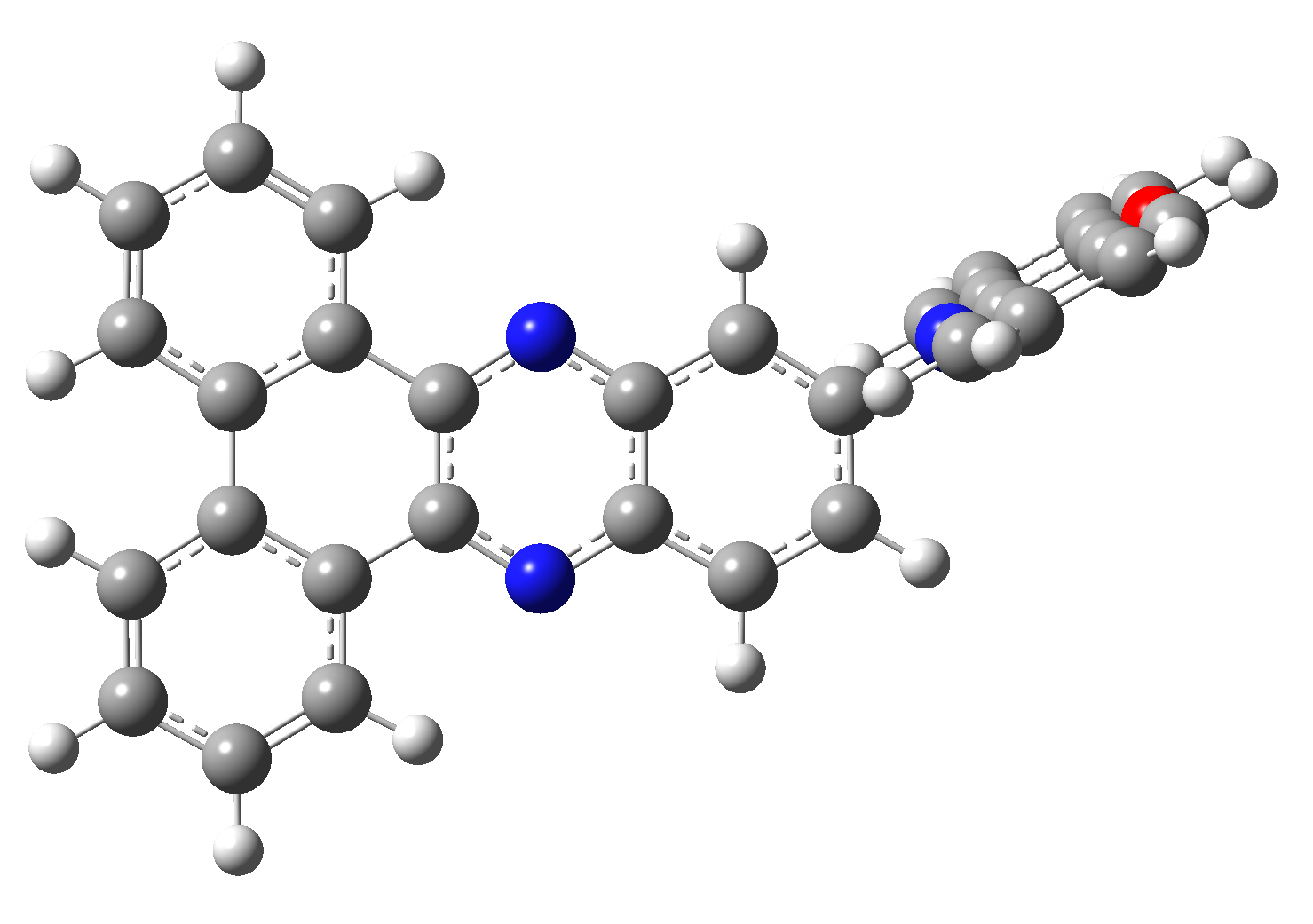 |
| Optimized geometry parameters | R_1_=1.4314Å;  θ=77.68˚; | R_1_=1.4669Å;  θ=90.07˚; |

**Table S2** Absorption and emission peak positions of compounds in different solvents. PXZ-DPPZ is not emissive in the solvents that the polarity is larger than Ethyl ether.

| solvents | *f*(*ε*,*n*) | CZP-DPPZ | | |
| --- | --- | --- | --- | --- |
|  |  | λ_a_  (nm) | λ_f_  (nm) | *v*_a_-*v*_f_ (cm^-1^) |
| Hexane | 0.0012 | 407 | 432 | 1422 |
| Triethylamine | 0.048 | 409 | 451 | 2277 |
| Isopropyl ether | 0.145 | 408 | 459 | 2723 |
| Ethyl ether | 0.167 | 408 | 468 | 3142 |
| Ethyl acetate | 0.200 | 409 | 498 | 4369 |
| Tetrahydrofuran | 0.210 | 411 | 502 | 4410 |
| Dimethyl formamide | 0.276 | 412 | 560 | 6414 |
| Acetone | 0.284 | 409 | 548 | 6201 |
| Acetonitrile | 0.305 | 408 | 559 | 6621 |

| solvents | *f*(*ε*,*n*) | TPA-DPPZ | | |
| --- | --- | --- | --- | --- |
|  |  | λ_a_  (nm) | λ_f_  (nm) | *v*_a_-*v*_f_ (cm^-1^) |
| Hexane | 0.0012 | 429 | 470 | 2033 |
| Triethylamine | 0.048 | 434 | 516 | 3661 |
| Isopropyl ether | 0.145 | 432 | 524 | 4064 |
| Ethyl ether | 0.167 | 431 | 530 | 4333 |
| Ethyl acetate | 0.200 | 432 | 557 | 5194 |
| Tetrahydrofuran | 0.210 | 436 | 564 | 5205 |
| Dichloromethane | 0.217 | 437 | 584 | 5760 |
| Dimethyl formamide | 0.276 | 440 | 608 | 6279 |
| Acetonitrile | 0.305 | 431 | 615 | 6941 |

| solvents | *f*(*ε*,*n*) | PXZ-DPPZ | | |
| --- | --- | --- | --- | --- |
|  |  | λ_a_  (nm) | λ_f_  (nm) | *v*_a_-*v*_f_ (cm^-1^) |
| Hexane | 0.0012 | 486 | 558 | 2654 |
| Triethylamine | 0.048 | 486 | 592 | 3684 |
| Isopropyl ether | 0.145 | 484 | 616 | 4427 |
| Ethyl ether | 0.167 | 477 | 632 | 4838 |
| Ethyl acetate | 0.200 | - | - | - |
| Tetrahydrofuran | 0.210 | - | - | - |
| Dimethyl formamide | 0.276 | - | - | - |
| Acetone | 0.284 | - | - | - |
| Acetonitrile | 0.305 | - | - | - |

**Table S3** Lifetimes of compounds in different solvents and doped film.

| Lifetime | CZP-DPPZ | TPA-DPPZ | PXZ-DPPZ |
| --- | --- | --- | --- |
| Hexane | 0.59 ns | 1.98 ns | 8.74 ns |
| Ether | 1.46 ns | 6.07 ns | - |
| THF | 4.44 ns | 7.37 ns | - |
| Doped film | 2.17 ns | 6.35 ns | 10.0 ns/1.3µs |

## Table S4. The radiative and non-radiative rate constants of compounds by MOMAP.

| Rate constant | CZP-DPPZ | TPA-DPPZ | PXZ-DPPZ |
| --- | --- | --- | --- |
| K_r_ | 7.32×10^6^ | 1.23×10^8^ | 1.82×10^3^ |
| K_nr_ | 2.12×10^13^ | 4.90×10^12^ | 6.40×10^11^ |

**Table S5.** The electrochemical and thermal properties of emissive materials.

| Compounds | T_d_ (^O^C) | HOMO (eV) | LUMO (eV) | Energy gap (eV) |
| --- | --- | --- | --- | --- |
| CZP-DPPZ | 454 | -5.58 | -3.21 | 2.37 |
| TPA-DPPZ | 434 | -5.26 | -3.17 | 2.09 |
| PXZ-DPPZ | 430 | -5.09 | -3.24 | 1.85 |
